# The role of the redox/miR-6855-3p/PRDX5A axis in reversing SLUG-mediated *BRCA2* silencing in breast cancer cells

**DOI:** 10.1186/s12964-019-0493-5

**Published:** 2020-01-27

**Authors:** Marshall Ellison, Mukul Mittal, Minu Chaudhuri, Gautam Chaudhuri, Smita Misra

**Affiliations:** 1 Department of Microbiology, Immunology, and Physiology, Meharry Medical College, Nashville, TN 37208 USA; 2 School of Graduate Studies and Research, Meharry Medical College, Nashville, TN 37208 USA; 3 Center for Women’s Health Research (CWHR), Meharry Medical College, Nashville, TN 37208 USA

**Keywords:** Breast cancer, BRCA2, PRDX5A, SLUG, miR6855-3p, Redox, de-silencing

## Abstract

**Background:**

We have previously shown that the zinc finger transcription repressor SNAI2 (SLUG) represses tumor suppressor *BRCA2*-expression in non-dividing cells by binding to the E2-box upstream of the transcription start site. However, it is unclear how proliferating breast cancer (BC) cells that has higher oxidation state, overcome this repression. In this study, we provide insight into the mechanism of de-silencing of BRCA2 gene expression by PRDX5A, which is the longest member of the peroxiredoxin5 family, in proliferating breast cancer cells.

**Methods:**

We used cell synchronization and DNA affinity pulldown to analyze PRDX5A binding to the *BRCA2* silencer. We used oxidative stress and microRNA (miRNA) treatments to study nuclear localization of PRDX5A and its impact on BRCA2-expression. We validated our findings using mutational, reporter assay, and immunofluorescence analyses.

**Results:**

Under oxidative stress, proliferating BC cells express PRDX5 isoform A (PRDX5A). In the nucleus, PRDX5A binds to the *BRCA2* silencer near the E2-box, displacing SLUG and enhancing *BRCA2*-expression. Nuclear PRDX5A is translated from the second AUG codon in frame to the first AUG codon in the *PRDX5A* transcript that retains all exons. Mutation of the first AUG increases nuclear localization of PRDX5A in MDA-MB-231 cells, but mutation of the second AUG decreases it. Increased mitronic hsa-miRNA-6855-3p levels under oxidative stress renders translation from the second AUG preferable. Mutational analysis using reporter assay uncovered a miR-6855-3p binding site between the first and second AUG codon in the *PRDX5A* transcript. miR-6855-3p mimic increases accumulation of nuclear PRDX5A and inhibits reporter gene translation.

**Conclusion:**

Oxidative stress increases miR-6855-3p expression and binding to the inter-AUG sequence of the *PRDX5A* transcript, promoting translation of nuclear PRDX5A. Nuclear PRDX5A relieves SLUG-mediated *BRCA2* silencing, resulting in increased *BRCA2*-expression.

**Graphical abstract:**

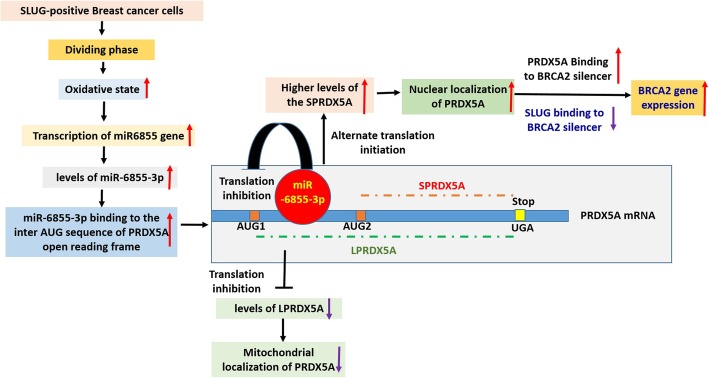

## Background

Since its discovery in 1995, tumor suppressor BRCA2 has become one of the most famous cancer-related genes [1]. BRCA2 is involved in various cellular functions, including maintenance of genomic stability during cell growth and proliferation through homologous recombination and DNA repair [2–4]. Hereditary or sporadic loss of BRCA2 function is associated with various cancers [3, 4] including breast [2–4], ovarian [5], esophageal [6] and prostate [7, 8] cancers. Dysregulated DNA repair and cell proliferation are critical to mammary tumorigenesis [9]. BRCA2-expression is tightly regulated during the cell cycle [10]. Levels of BRCA2 mRNA and protein should commensurate with the status of the cells; they are upregulated in proliferating breast cancer (BC) cells but repressed in non-dividing (G0/G1) cells due to its toxicity [10–12]. Although cell cycle-dependent regulation of BRCA2-expression is well established, its mechanism is not well understood. We have previously shown that BRCA2-expression is silenced by SLUG in SLUG-positive BC cells [13], and by ZAR2 when SLUG is absent [14]. SLUG silences BRCA2-expression by binding to the E2-box within the 221-bp silencer region located 700-bp upstream of the transcription start site [13]. The E2-box resides within the 81-bp inter-Alu sequence between two Alu repeats [13, 15].

Alu sequences are short repetitive DNA elements dispersed throughout the primate genome [16]. Alu RNAs are transcribed by RNA polymerase III (polIII) [17, 18]. The accumulation and processivity of Alu RNAs have been associated with various cancers [19, 20] and age-related macular degeneration [21]. Alu co-repressor1 (ACR1), also known as peroxiredoxin 5 (PRDX5), represses RNA polIII-mediated Alu RNA transcription [22].

Peroxiredoxins form a superfamily of six (human) thiol-dependent peroxidases that reduce hydrogen peroxide (H_2_O_2_), alkyl hydroperoxides, and peroxynitrites [23, 24]. They possess a conserved peroxidatic Cys (C_P_) at the N-terminus. During the peroxidase reaction, the C_P_ attacks the O-O bond of the peroxide and is subsequently oxidized to sulfenic acid, which is then restored to C_P_ during the resolution step. Based on this resolution mechanism and either the presence or absence of a resolving Cys (C_R_) at the C-terminus of the enzyme, peroxiredoxins are classified into three subfamilies: 1-Cys, typical 2-Cys, and atypical 2-Cys [23, 24]. PRDX5 is an atypical 2-Cys peroxiredoxin that possesses a C_R_ within the same polypeptide chain. Its C_R_ reacts with its C_P_ to form an intramolecular disulfide bond [25]. In addition to their antioxidant activity, peroxiredoxins also appear to influence signaling pathways that have a redox-dependent component [24, 26]. PRDX5 interacts with peroxisome-receptor-1 and exerts an antioxidant function in different tissues under normal conditions and during inflammatory processes [27]. As per NCBI Gene-database, human *PRDX5* resides at chromosome 11, and has four splice variants generated from the same transcript, with transcription start site at 64318088-bp. PRDX5A is the longest isoform that retains all six exons. PRDX5B lacks exon 3, PRDX5C lacks exon 2 and 3, and PRDX5D lacks exon 2. The use of alternative transcription start sites and splice-variants is thought to yield transcript variants that generate PRDX5 isoforms that localize to either the mitochondria, peroxisome/cytoplasm or nucleus [27]. However, exact mechanism of biogenesis for the nuclear form of PRDX5 is not known.

Here, we elucidate how PRDX5A reverses SLUG-mediated repression of BRAC2 expression in dividing SLUG-positive BC cells. In this study, we found that nuclear PRDX5A is translated from the second in-frame AUG codon in the open reading frame (ORF) of the *PRDX5A* mRNA, yielding the short (S) isoform (SPRDX5A) that lacks mitochondrial localization signal. This translation is mediated by a unique, redox-induced mitronic miRNA hsa-miR-6855-3p located in intron 13 of *USP21 for USP20 and P22*. We demonstrate that in an oxidizing environment during cell division, miR-6855-3p levels are upregulated. Using artificial constructs, we show that treating the cells with miR6855-3p leads to increased nuclear levels of SPRDX5A, which reverses SLUG-induced *BRCA2* silencing *via* binding to and displacing SLUG from the *BRCA2* silencer. Our study highlights the cell cycle-dependent regulation of BRCA2-expression and a novel mechanism in which miR6855-3p determines where translational begins on PRDX5A mRNA.

## Methods

### Reagents and antibodies

Antibodies against PRDX5 (BD Biosciences), BRCA2 (Cell Signaling Technology), fibrillarin, GSK3β, SLUG, HSP90, and VDAC1 (Santa Cruz Biotechnology), β-actin, GAPDH, FLAG (M2), (Sigma), and HRP-conjugated secondary antibodies against mouse and rabbit (GE) were used. H_2_O_2_, sulforaphane (SFP), ter-butyl hydrogen peroxide (tBHP), MG132, 2′,7′-dichlorodihydrofluorescein diacetate (DCFDA), cell-lytic reagent, β-mercaptoethanol, and protease inhibitor cocktail were from Sigma. All primers, restriction enzymes, and Trizol came from Life Technologies. For miRNA isolation we used miRNesay kit from Qiagen. Plasmid DNA was isolated using the plasmid DNA isolation kit (Qiagen) also 2X TaqDNA Mix (Qiagen) was used for amplification. For amplification of the open reading frame (ORF) Pfu-Turbo (Agilent) was used. The primers used in this study are listed in Additional file 1: Table S1.

### Cell culture and synchronization

All breast cancer cell lines were procured from American Type Culture Collection (ATCC, Manassas, VA) and cultured as described [13–15]. Cell lines authentication are routinely performed following instructions in ATCC Bulletin 8. Cell synchronization was performed using serum starvation as described previously [13, 14]. Briefly, cells were seeded at 30–50% confluency in complete growth media with 10% FBS and incubated at 37 °C in a humidified chamber with 5% CO_2_. After 16–18 h, the cells were washed, and the complete media was replaced with the starvation media (RPMI 1640, phenol red free, 0% fetal bovine serum). The cells were starved for 36 h to arrest them at G_0_. The cells were released by replacing the starvation media with complete media containing 10% FBS. The cells were then incubated for 20 h before harvesting the dividing population. Cell cycle progression was monitored by flow cytometry analysis of propidium iodide-stained cells [13]. Cells were treated with 20 μM 2′,7′ –dichlorofluorescin diacetate (DCFDA) for 30 min and fluorescence was measured to monitor the redox state of the cells. DCFDA is a cell permeable fluorescent dye. It is taken up by the live cells and after diffusion into the cell, DCFDA is deacetylated by cellular esterases to a non-fluorescent compound, which is later oxidized by reactive oxygen species into 2′, 7′ –dichlorofluorescein (DCF). DCF is a highly fluorescent green compound which can be detected by fluorescence excitation 495 nM. The higher the reactive oxygen species the more green are the cells. For transfection and synchronization experiments, cells were transfected with plasmids of interest and allowed to recover for 6 h in complete growth media before starvation [14].

### *BRCA2* promoter-silencer constructs, transfection, and dual luciferase assay

Human *BRCA2* promoter-silencer (− 921 to + 301) was amplified from genomic DNA isolated from BT549 BC cells using primers P1 and P3 (Additional file 1: Table S1) [13]. The amplified PCR product was cloned into pCRIV-Topo (Invitrogen) and its sequence was verified using primers T7 and T3. The promoter-silencer insert was digested out of the recombinant plasmid with EcoRI and subcloned into pRL-Null (Promega). Clones with the insert in the reverse orientation with respect to the T7 RNA polymerase promoter (pRL-PS) was selected and used for reporter assays to study the effect of the silencer on *BRCA2* promoter activity in SLUG-positive BC cells. Transient transfections were performed in 24-well plates using Lipofectamine 2000 (Invitrogen) with pRL-PS (0.8 μg) and pGL3 firefly luciferase control vector (0.08 μg) (Promega). Protein lysates were prepared from the cells, and luciferase activity was measured as described previously [13]. *Renilla* luciferase activity was normalized to the firefly luciferase activity and presented as a ratio (relative light units). Transfected cells were left to recuperate for 6 h before cell synchronization and H_2_O_2_ treatment. Protein concentrations of the extracts, when needed, were determined using RC-DC reagents and protocol from Bio-Rad.

### Recombinant protein expression in BC cells

For ectopic expression of PRDX5 in BC cells the coding sequence for human *PRDX5* was amplified from RNA isolated from BT549 cells using *PRDX5*-specific primers P4 and P5 (Additional file 1: Table S1) without the stop codon [14, 28]. The amplified cDNAs for *PRDX5A* (642 bp), *PRDX5B* (510 bp), and *PRDX5C* (375 bp) were gel-purified using Qiagen gel extraction kit and cloned into pCRIV-Topo. The cDNA inserts were sequence-verified, digested with ClaI/BamHI, and cloned into p3XFLAG-CMV-14 (Sigma) to obtain the C-terminal FLAG-tagged PRDX5A, PRDX5B, and PRDX5C constructs. For the cloning of PRDX5A into pZsGreen_N1 (Clontech), the PRDX5A cDNA was amplified using primers P6 and P7 (Additional file 1: Table S1) and subcloned between the BamHI/HindIII restriction sites to generate long form PRDX5A (LPRDX5A).

### Site-directed mutagenesis

The two in-frame ATG codons were mutated in the pre-cloned PRDX5A-Flag-tagged construct as described briefly. PCR-based site-directed mutagenesis was performed using the QuikChange site-directed mutagenesis kit (Stratagene) to generate the ATG1-mutant and ATG2-mutant PRDX5A constructs [13]. The start codon was mutated from 5′-ATG-3′ to 5′-GCC-3′ and second start codon was mutated from 5′-ATG-3′ to 5′-GCG-3′ (sense strand) using overlapping primers (P8/P9 and P10/P11 (Additional file 1: Table S1) for first and second ATG, respectively). After sequence verification, the mutant ORF was re-cloned into p3XFLAG-CMV-14 (Sigma) as described earlier. The mutations ablate the function of the start codon without disrupting the reading frame.

### Inter-AUG reporter assay

Wild type inter-AUG sequence was amplified from the pre-cloned *PRDX5A* ORF. For mutation the inter-AUG sequence was amplified from the pre cloned *PRDX5A* ORF using primers P12 and P13 (Additional file 1: Table S1) and cloned into pCRIV-Topo. Splice overlap extension (SOE) PCR [29] was performed using the cloned *PRDX5A* ORF as template to mutate the entire putative miR-6855-3p binding site within the inter-AUG sequence. Briefly, two separate reactions were performed in the first PCR using primers P12 and P15 (Additional file 1: Table S1) to create amplicon T1 and primers P13 and P14 to generate amplicon T2. T1 and T2 have complementary overlapping sequences created using primers P14 and P15. In the second PCR, T1 and T2 served as templates for PCR using primers P12 and P13 to create the mutated inter-AUG sequence that lacks the miR-6855-3p binding site. After sequence verification, the wildtype and mutant inter-AUG inserts were separately subcloned in the pMIR-REPORT luciferase vector (Clontech) between the Spe1/HindIII restriction sites. To perform the Dual luciferase assay pNLTK (Promega) was used as a source of normalizing nanoLuc (modified *Renilla*) luciferase. In 24 well platform, co-transfection of BC cells was done with pMIR-REPORT (1 μg) expressing either the wildtype or mutant inter-AUG, pNLTK (0.1 μg) (Promega) and miRNA6855-3p mimic (Ambion) was performed using TurboFect. Transfected cells were cultured for 24 h, after which Dual luciferase assay was done using NanoGlo Dual luciferase assay kit (Promega) as per the manufactures instruction. Firefly luciferase activity from pMIR-Report was measured and normalized to NanoLuc® luciferase from pNLTK activity and presented as a ratio (relative light units, RLU).

### Subcellular fraction

Nuclear and cytoplasmic fractions were obtained using NE-PER nuclear and cytoplasmic protein extraction reagents (Thermo Fisher Scientific). The quality of the fractions was determined by standard assays [28].

### DNA affinity pulldown

Biosynthetically labeled, ^35^S-methionine nuclear extracts were used for DNA affinity pulldown for the *BRCA2* silencer as described previously [13].

### Electrophoretic mobility shift assay (EMSA)

5′-biotynyated silencer (221 bp) was amplified by PCR using 5′-biotynylated primers P2/P3 (Additional file 1: Table S1). EMSA was performed with the purified silencer DNA and nuclear-enriched fractions from BT549 cells as described previously [13]. For supershift assay anti-PRDX5 antibody (BD-Bioscience) was used.

### PRDX5 and SLUG knockdown

*PRDX5* siRNA pool and control siRNA were procured from Santa Cruz Biotechnology. SLUG siRNA was procured from Ambion, lnc, Huston, TX (catalog# 4390824). Cells were plated in six-well plates (2.5 X 10^5^/well) on Day 0. On Day 1, the cells were transfected with 100 nM of either SLUG, *PRDX5* or control siRNAs using Lipofectamine 2000. Cells were harvested 48 h post-transfection and processed for subsequent experiments [28].

### Quantitative reverse transcription PCR (qRT-PCR)

RNA isolation and quantitation were done as mentioned previously [28]. Total RNA was extracted from BC cells using Trizol. The isolated RNA was treated with DNase to remove contaminating DNA. Reverse transcription was performed using the Iscript cDNA synthesis kit (BioRad). The cDNA (equivalent to 50 ng of total RNA/reaction) was incubated with SYBR Green and respective primers for 40 cycles at 95 °C and 1 min at 55 °C on a Bio-Rad real-time PCR system. Fluorescence measurement was recorded at 55 °C after each cycle. After the final cycle, a melting curve analysis was conducted within the range of 55–95 °C for all samples. Relative gene expression was quantified using β-actin and GAPDH as internal controls. The threshold cycle and 2^-ΔΔCt^ methods were used to calculate the relative amounts of the target RNA. The experiments were repeated at least three times in triplicate. For total miRNA quantitation, miRNA-enriched fraction was obtained with column capture using the miRNeasy kit (Qaigen). The miRNA was converted to cDNA using universal primers and reagents from miScript IIRT kit (Qiagen). The miScript SYBR Green kit (Qiagen) was used to quantify miR6855-3p. RNU6 and 15a amplification was used for normalization. Universal primers, RNU6 and 15a primers were from Qiagen.

### Immunoblot analysis

Whole cell lysates were prepared by homogenization in RIPA lysis buffer (50 mM Tris-HCl, pH 7.4; 1% NP-40; 0.25% sodium deoxycholate; 150 mM NaCl; 1 mM EDTA; 1X protease inhibitor cocktail, 1X phosphatase inhibitor), sonication, and incubation at 4 °C for 20 min, followed by centrifugation at 12000×g at 4 °C for 10 min. Extracts containing equal amounts of proteins were separated by SDS-PAGE on 10–12% polyacrylamide gels and transferred to nitrocellulose membranes. The membranes were probed with primary antibodies against BRCA2 (1:000), HSP90 (1:1000), VDAC1 (1:1000), GSK3β (1:1000), Fibrillarin (1:500), PRDX5 (1:100), GAPDH (1:5000), β-actin (1:3000), and FLAG (1:500). HRP-conjugated bovine secondary antibodies (GE healthcare) were used for visualization. Chemiluminescence was detected using the ECL substrate from Thermofisher [28].

### Immunofluorescence analysis

The subcellular localization of the C-terminal FLAG-tagged PRDX5A was analyzed by confocal microscopy using anti-FLAG antibody conjugated with Cy3 (Sigma) as previously described [14, 28]. For experiments using the pZS-Green-LPRDX5A constructs, BC cells were grown to ~ 80% confluency on glass coverslips in 24-well plates and co-transfected with different concentrations of miR6855-3p miRNA mimic (Ambion) (0, 15 and 30 pmole) and 1 μg/well pZsGreen-LPRDX5A using Lipofectamine 2000. After 24 h, the transfected cells were incubated with MitoTracker® Red CMXRos (Invitrogen) to stain the mitochondria. The coverslips were then mounted with ProLongTM Diamond Antifade Mountant containing DAPI (Invitrogen) to stain the nuclei blue. The stained, fixed cells were visualized and photographed using a Nikon TE2000-U C1 confocal laser-scanning microscope. The laser gain for each color is as follows: EGFP at 100, DAPI at 105, and TRITC at 115; the offset was − 7 throughout. Bezier tool was used to highlight individual cells and Pearson coefficients [30] for colocalization were measured using NES analysis tool. For analysis n for Control = 8, 15 pmole = 6 and 30pmole = 13.

### PRDX5A MLS (mitochondrial localization signal) and NLS (nuclear localization signal) construct and analysis

The MLS of human PRDX5A was amplified from pre-cloned *PRDX5A* ORF using primers P16 and P17 (Additional file 1: Table S1). The NLS was amplified using primers P18 and P19 (Additional file 1: Table S1) from the pre-cloned PRDX5A ORF. The amplified cDNA was sequence-verified as described earlier, digested with BamHI/HindIII, and cloned into ptdTomato-N1 (Clontech). BC cells were grown to ~ 80% confluency on glass coverslips in 24-well plates and transfected with 1 μg/well of the ptdTomato-N1-MLS plasmid using Lipofectamine 2000. After 24 h, the coverslips were mounted with ProLongTM Diamond Antifade Mountant. DAPI was used to stain the nuclei blue. Images were captured using confocal microscope as mentioned previously [28].

### Quantitative (q) chromatin immunoprecipitation (ChIP) assay

ChIP assay was performed as described previously [13, 14, 28, 31, 32]. For each assay, 10^6^ cells were used. Chromatin pulldown was performed using 2.4 μg of the target antibody coupled to magnetic beads. The reaction mixture was incubated overnight with the sonicated chromatin at absorbance A_260_ = 2 at 4 °C [32]. Following pulldown, washing, decrosslinking, and proteinase K treatment, the DNA product was column-purified using the Qiagen PCR purification kit. Mouse IgG was used to pull down PRDX5, while rabbit IgG was used to pull down SLUG for normalization. End-point or real-time PCR quantification of the purified DNA product was performed following standard protocols using SYBR Green dye (Bio-Rad) with primers P2 and P3 (Additional file 1: Table S1) to evaluate the binding of PRDX5 or SLUG at the *BRCA2* silencer region. In qChIP assay, fold change over control samples was calculated using *Ct*, Δ*Ct*, and ΔΔ*Ct* values. Ten percent of the total DNA used for the pulldown was used as input control for normalization [13, 14, 28, 31, 32].

### *USP21 for USP20 and P22* and miR6855 promoter constructs and luciferase activity

The non-coding, putative promoter regions of human *USP21 for USP20 and P22* (Sequence ID: NC_000009.12 from 129,834,543 to 129,835,506) and miR6855 miR6855 (Sequence ID: NC_000009.12 from 129,868,553 to 129,869,604) were amplified from genomic DNA isolated from BT549 cells using primers P21 for USP20 and P22/P21 for *USP21 for USP20 and P22* and P22/P23 for miR6855, respectively [13–15]. The details for primers are in Additional file 1: Table S1. The amplified DNA (*USP21 for USP20 and P22*: 964 bp; miR6855: 1052 bp) was cloned into pCRIV-Topo and sequence-verified using primers T3 and T7. Sequence-verified inserts were subcloned into pNL1.1 (Promega) at the XhoI/HindIII restriction sites for *USP21 for USP20 and P22* and the HindIII restriction site for miR6855 [13–15]. For luciferase activity measurement, BC cells were grown to ~ 80% confluency in 24-well plates and co-transfected with 0.8 μg of either the pNL1.1-promoter construct or pNLTK and 0.08 μg of pGL3 control using Lipofectamine 2000. After 18 h, the cells were treated with 10 mM SFP for 1 h and then fed with fresh media for a 5-h recovery. The cells were then lysed using passive lysis buffer (Promega), and a dual luciferase assay was performed using the Nano-Glo Dual-Luciferase assay kit (Promega). NanoLuc luciferase activity was normalized to firefly luciferase activity and presented as a ratio (relative light units, RLU).

### Statistical analysis

Each experiment was repeated at least three times. Results were expressed as means ±SEM. Statistical analysis was performed using GraphPad Prism and Microsoft Excel. *P* values were calculated using the two-sided Student’s *t*-test (paired or unpaired, as appropriate) and analysis of variance for significance. *P* values < 0.05 and < 0.01 were considered statistically significant.

## Results

### The human *BRCA2* gene silencer region contains a PRDX5 binding site

**Fig. 1:**
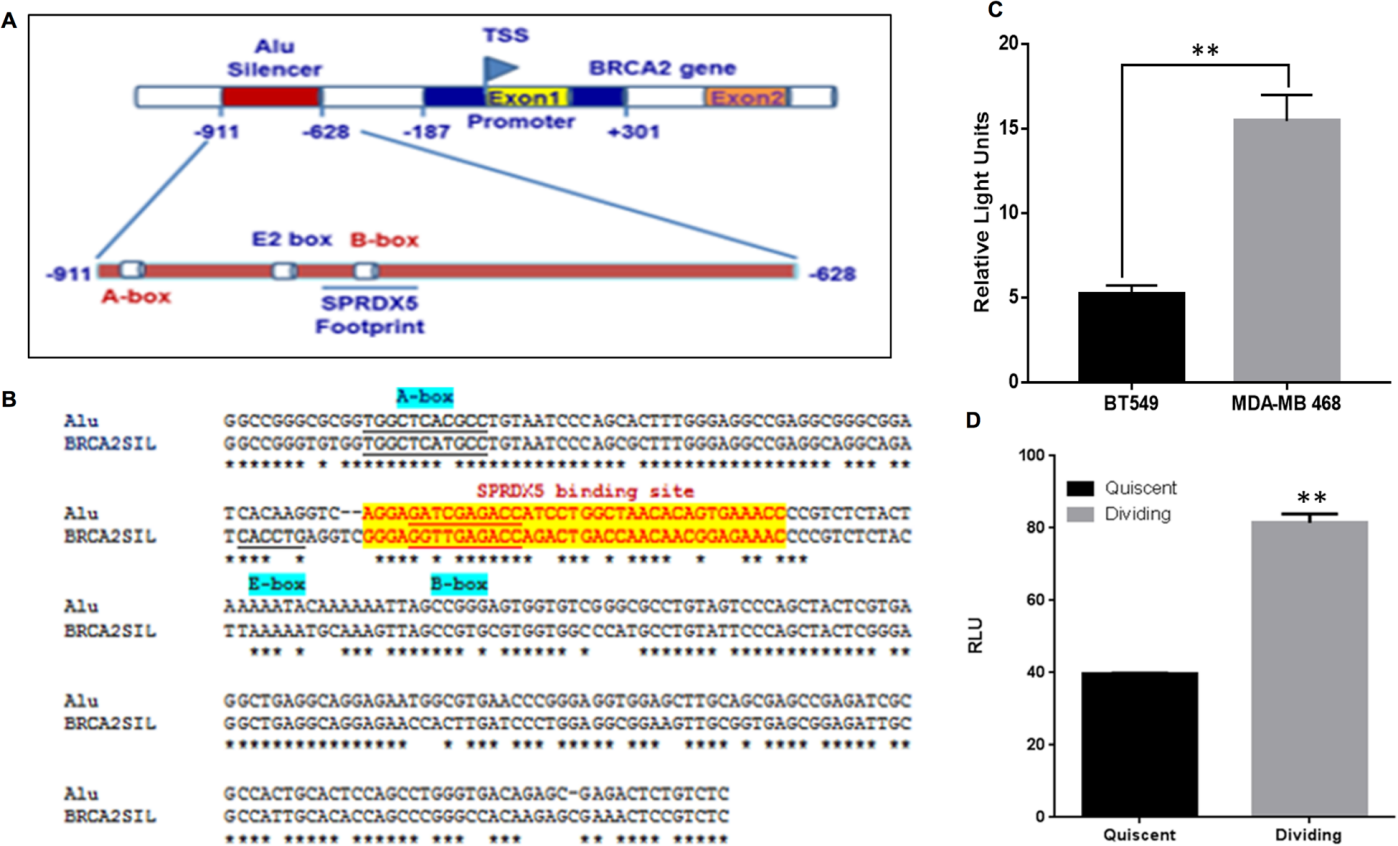
Human *BRCA2* gene silencer is de-silenced in dividing SLUG-positive BT549 cells. **a** A schematic of the human *BRCA2* promoter and silencer. A detailed illustration of the silencer shows the E2-box (SLUG binding site) and putative PRDX5 binding site (underlined). The A-box and B-box represent sequences required for translation initiation by RNA polIII. The numbers shown are with respect to the *BRCA2*-transcription start sites. **b** CLUSTAL-Omega-(1.2.4) sequence alignment of the human *BRCA2* silencer with that of full-length Alu element. The putative E2-box, A-box, and B-box are labeled. The putative SPRDX5 binding site is highlighted. **c** Activity of the *BRCA2* promoter in the presence of the silencer in SLUG-positive BT549 and SLUG-negative MDA-MB-468 cells. **d** Activity of the *BRCA2* promoter in the presence of the silencer in quiescent (non-dividing) and dividing SLUG-positive MDA-MB-231 cells. Data are presented as mean ± SE (*n* = 6). RLU, relative light units. The difference between the luciferase activity in the quiescent and dividing cells is statistically significant (*p* < 0.001)

Previously, we have uncovered a 221-bp silencer sequence upstream (− 701 to − 921 bp) of human *BRCA2 gene transcription start site* [15]. It consists of an E2-box flanked by Alu sequences (Fig. 1a, b). We have shown that SLUG binds to the E2-box to repress *BRCA2*-expression in SLUG-positive cells [13]. Detailed analysis of the *BRCA2* silencer region revealed a PRDX5 binding site within the Alu sequences, designated as “SPRDX5A footprint” in Fig. 1a, b. Using the reporter gene assay, we found that the silencer was more effective in SLUG-positive BT549 cells than in SLUG-negative MDA-MB-468 BC cells (Fig. 1c). We also have observed that human *BRCA2* gene promoter (− 187 to + 310) [14] construct lacking the silencer were not inhibited by the presence of SLUG as was evident by the similar luciferase activity in the SLUG-knockdown BT549 cells compared to control cells (Additional file 1: Figure S1A and S1B). While *BRCA2* gene promoter activity is significantly inhibited by the presence of SLUG and the silencer region. (Additional file 1: Figure S1B, 0 mM H_2_O_2_). Furthermore, Loss of SLUG *via* siRNA mediated knock-down in BT549 cells increased the BRCA2 promoter activity from the pRL-PS constructs compared to control cells by 2-fold (Additional file 1: Figure S1B, 0 mM H_2_O_2_). Interestingly, we found that the *BRCA2* promoter activity in the presence of the silencer was increased two-fold in dividing compared to quiescent BT549 cells (Fig. 1d). In agreement with published reports, we verified that dividing BC cells had a higher oxidative state than quiescent cells by staining the cells with DCFDA, which detects reactive oxygen species (Additional file 1: Figure S2).

### Human PRDX5 consists of multiple splice variants and isoforms

Through end-point RT-PCR analysis, of BC cells we detected three distinct cDNA products in all BC cell lines examined (Additional file 1: Figure S3a). However, we could not detect the transcript generated from the alternative transcription start site. Furthermore, we could only detect one protein band at ~ 18 kDa in all the cells examined (Additional file 1: Figure S3b). The predicted size of PRDX5A is ~ 24 kDa. It is possible that upon translocation to the mitochondria, the protein is reduced to ~ 18 kDa in size after MLS removal. Through subcellular fractionation and immune-detection using anti-PRDX5 antibody against the C-terminal of the protein, we observed that the size of nuclear PRDX5A is the same as that of mitochondrial PRDX5A (Additional file 1: Figure S3c). We have observed that the PRDX5B and PRDX5C isoforms are unstable and degraded by the proteasome when expressed ectopically (Additional file 1: Figure S3d). The ORF of *PRDX5A* also has two in-frame AUG codons in exon 1. Translation from the second AUG would yield a smaller protein (~ 18 kDa) that lacks the MLS. Because there is no basis to expect reverse protein translocation through the mitochondrial membrane, we hypothesize that all nuclear PRDX5 should originate from translation of the *PRDX5A* mRNA from the second ATG.

### PRDX5A accumulates in the nucleus during cell division and de-silences BRCA2-expression by binding to its promoter

**Fig. 2:**
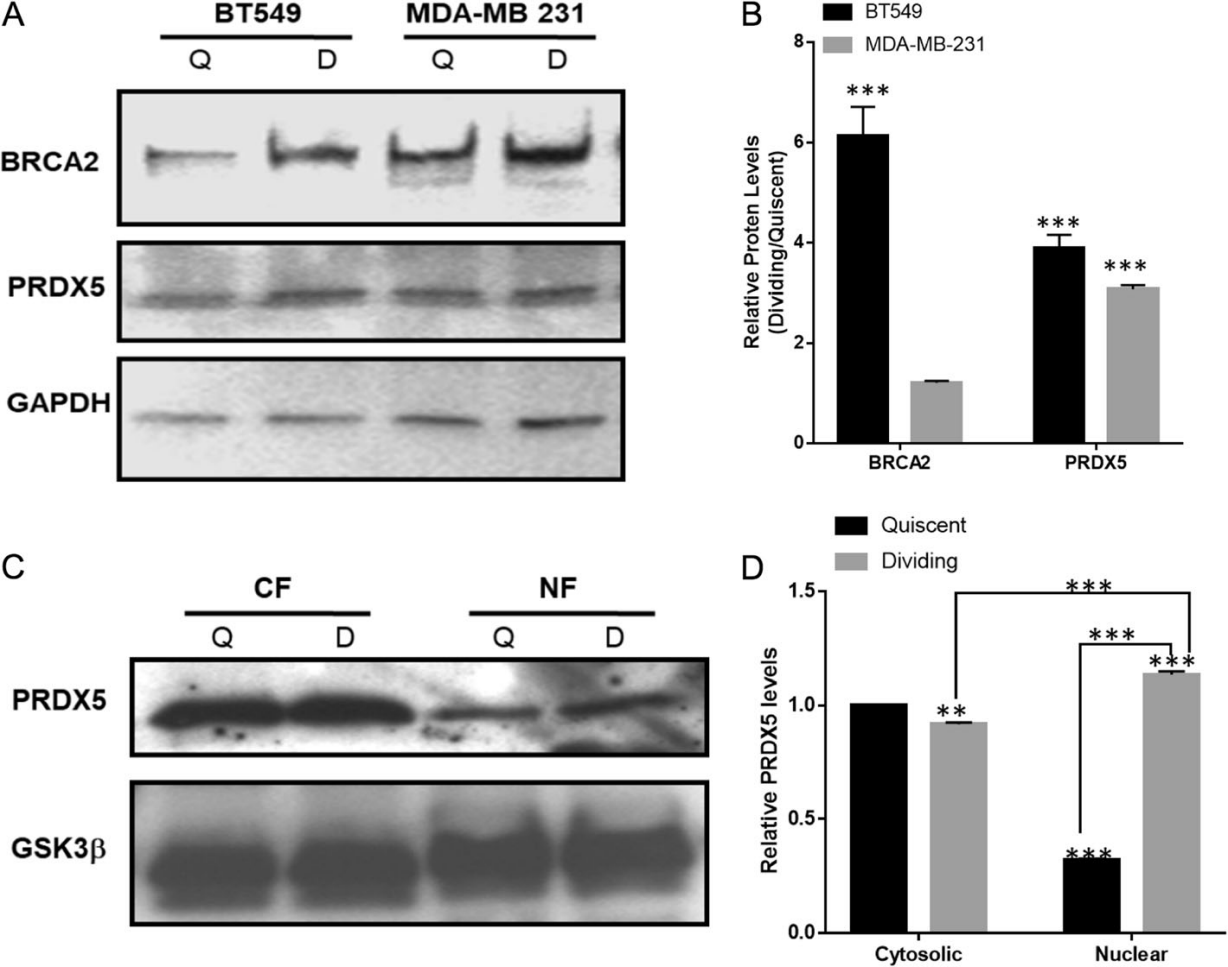
Expression and nuclear localization of PRDX5 increases in dividing cells. **a** Immunoblots of BRCA2 and PRDX5 in quiescent and dividing BC cells. Lysates (30 μg of protein/lane) were resolved on a 5–12% SDS-PAGE. GAPDH served as the loading control. **b** Densitometry of western blots such as those in **a** to evaluate the relative levels of BRCA2 and PRDX5 in dividing and quiescent cells. Results are mean ± SE (*n* = 3). *** indicates statistical significance, *p* < 0.0001. **c** Analysis of PRDX5 in the cytosolic and nuclear fractions (CF and NF, respectively) by western blotting. CF and NF were isolated from synchronized BT549 cells at the quiescent (Q) and dividing (D) stages. GSK3β served as a normalizing control since it is equally distributed in both fractions. **d** Densitometry of western blots such as those in **c** showing relative levels of nuclear and cytosolic PRDX5 in quiescent and dividing cells compared to levels of cytosolic PRDX5 in quiescent cells. Results are mean ± SE (*n* = 3). The difference in PRDX5A levels is statistically significant (*p* < 0.001)

To understand how PRDX5A reverses the silencing of BRCA2-expression, we first assessed the expression levels of both proteins in quiescent and dividing SLUG-positive BT549 and MDA-MB-231 cells. We observed six-fold increase in BRCA2-expression in dividing compared to quiescent BT549 cells (Fig. 2a, b). The increase was approximately two-fold in dividing MDA-MB-231 cells (Fig. 2a, b). The difference in fold increase between the two cell lines could be due to different levels of de-silencing or other genetic factors. In addition, PRDX5A expression also increased by about three- to four-fold in dividing cells compared to resting cells for both cell lines (Fig. 2a, b). To investigate the distribution of PRDX5A in the nucleus and cytosol of quiescent and dividing cells, we performed cellular fractionation followed by immunoblot analysis. We found that the levels of nuclear PRDX5A was higher in dividing cells than in quiescent cells (Fig. 2a, b). GSK3β, which is equally distributed in the cytosol and nucleus, served as the loading control (Fig. 2c, d).

**Fig. 3:**
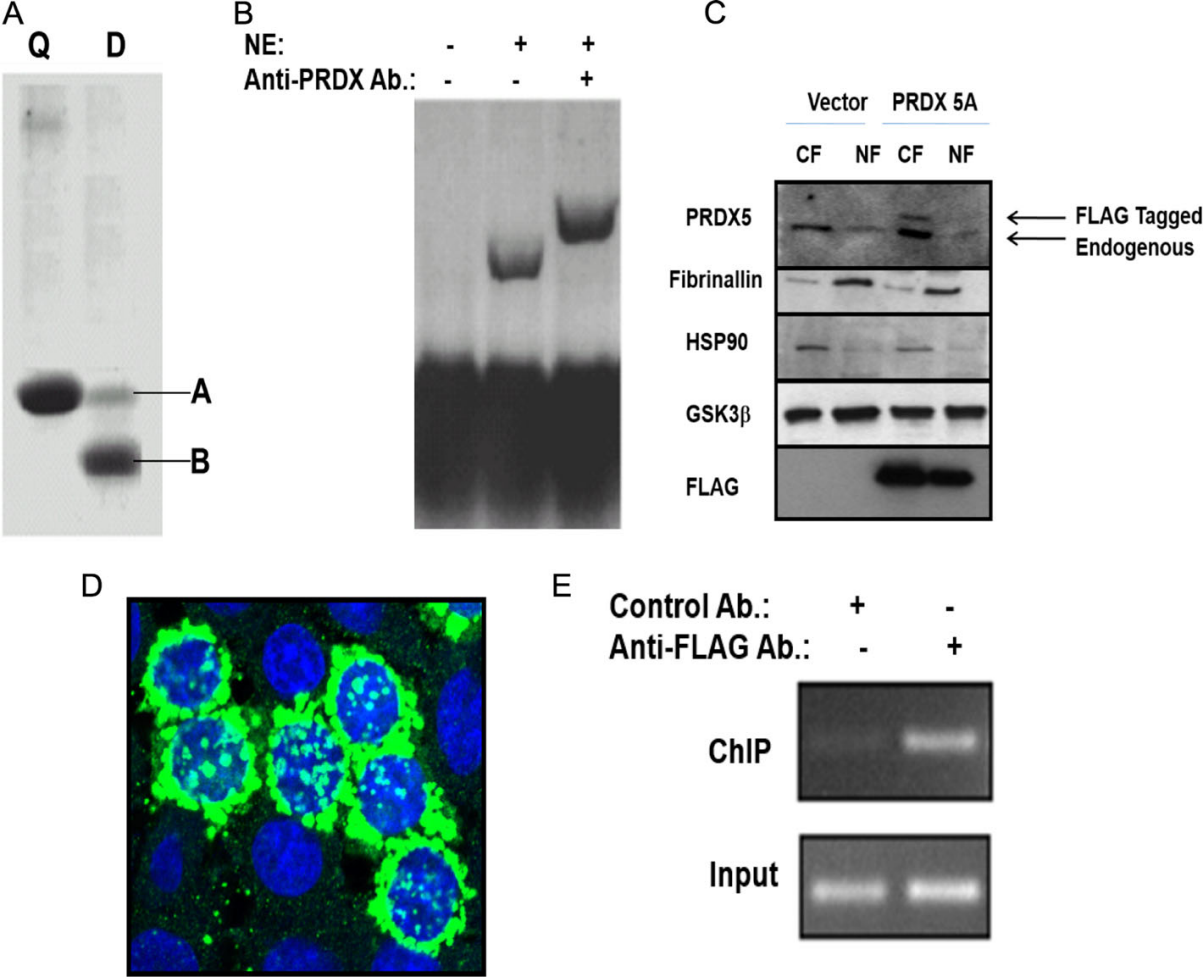
Nuclear PRDX5 binds to the *BRCA2* silencer in dividing cells. **a** An autoradiogram from DNA affinity purification using ^35^S-methionine-labeled nuclear extracts of quiescent (Q) and dividing (D) BT549 cells to detect proteins that bind to the *BRCA2* silencer. Band A (~ 29 kDa) corresponds to SLUG and band B (~ 18 kDa) corresponds to PRDX5. **b** A supershift assay showing PRDX5 binding to the *BRCA2* silencer. The 5′-biotynylated silencer DNA (221-bp) served as a probe. The probe was incubated with unlabeled nuclear extract (NE) from dividing BT549 cells. PRDX5 antibody was used to verify PRDX5 binding to the silencer probe. **c** Validation of the expression of C-terminal FLAG-tagged PRDX5A in BT549 cells. Transient transfection was performed using either the vector (p3XFLAG-CMV14) alone or p3XFLAG-CMV14-PRDX5A for 48 h before the isolation of the cytosolic (CF) and nuclear (NF) fractions. Fibrillarin, HSP90, and GSK3β served as a nuclear marker, a cytosolic marker, and the loading control, respectively. **d** Immunofluorescence analysis with anti-FLAG antibody (green) showing puncta of C-terminal FLAG-tagged PRDX5A in the nucleus and cytoplasm of the transiently transfected cells. The nuclei were stained blue with DAPI. **e** A ChIP assay showing in-vivo binding of PRDX5A at the silencer in breast cells transiently expressing FLAG-tagged PRDX5A. Anti-FLAG antibody was used to pulldown the protein. Anti-mouse IgG was used as a control antibody. The lower panel shows the amplification of input DNA prior to immunoprecipitation

To assess the binding of SLUG and PRDX5 to the *BRCA2*-silencer, we performed a DNA affinity pulldown assay using nuclear extracts from quiescent and dividing cells (Fig. 3a). We found that while the silencer pulled down SLUG (~ 29 kDa) from the nuclear extracts of quiescent cells, its binding to the silencer was significantly reduced in the nuclear extracts from dividing cells (Fig. 3a), suggesting that a de-silencing mechanism exists. Interestingly, we observed a smaller protein of ~ 18 kDa in size being pulled out from the dividing cells (Fig. 3a). As the *BRCA2* silencer possesses a PRDX5 binding site, our observations indicate that while SLUG binds the silencer in quiescent cells, the redox-responsive nuclear PRDX5A perhaps binds the silencer in dividing cells, which experience higher oxidative stress. To determine if this 18-kDa protein is PRDX5, we performed an EMSA in the presence/absence of anti-PRDX5 antibody. EMSA using the *BRCA2* silencer DNA and radiolabeled nuclear proteins obtained from dividing cells showed a clear shift of DNA band due to reduced mobility, indicating protein binding (Fig. 3b). Addition of anti-PRDX5A antibody to the reaction mixture shifted this band further, confirming that the bound protein was PRDX5A (Fig. 3b). To validate PRDX5A binding on the *BRCA2* silencer in-vivo, we performed ChIP analysis using breast cells by expressing C-terminal FLAG-tagged-PRDX5A in these cells (Fig. 3c, d). Immunoblot analysis of the cytosolic and nuclear fractions from these cells showed that FLAG-tagged-PRDX5A was present in both fractions from the PRDX5A-FLAG-transfected cells but not in the vector control cells (Fig. 3c). As expected, endogenous PRDX5A was detected in both the PRDX5A-FLAG-transfected and the vector-transfected cells (Fig. 3c). In-situ immunofluorescence analysis of the PRDX5A-FLAG-transfected cells using the anti-FLAG antibody showed the presence of PRDX5A-FLAG in the cytosol and the nucleus (Fig. 3d). For ChIP analysis, immuno-pulldown of the chromatin fragment by anti-FLAG antibody followed by end-point PCR using primers specific for the *BRCA2* silencer revealed PRDX5A binding to the silencer in BC cells (Fig. 3e). Taken together, our data show that PRDX5A localizes to the nucleus and binds to the *BRCA2* silencer. This binding may in turn facilitate BRCA2-expression.

### Oxidative stress-induced de-silencing of *BRCA2*-expression correlates with nuclear accumulation of and silencer binding by PRDX5A

**Fig. 4:**
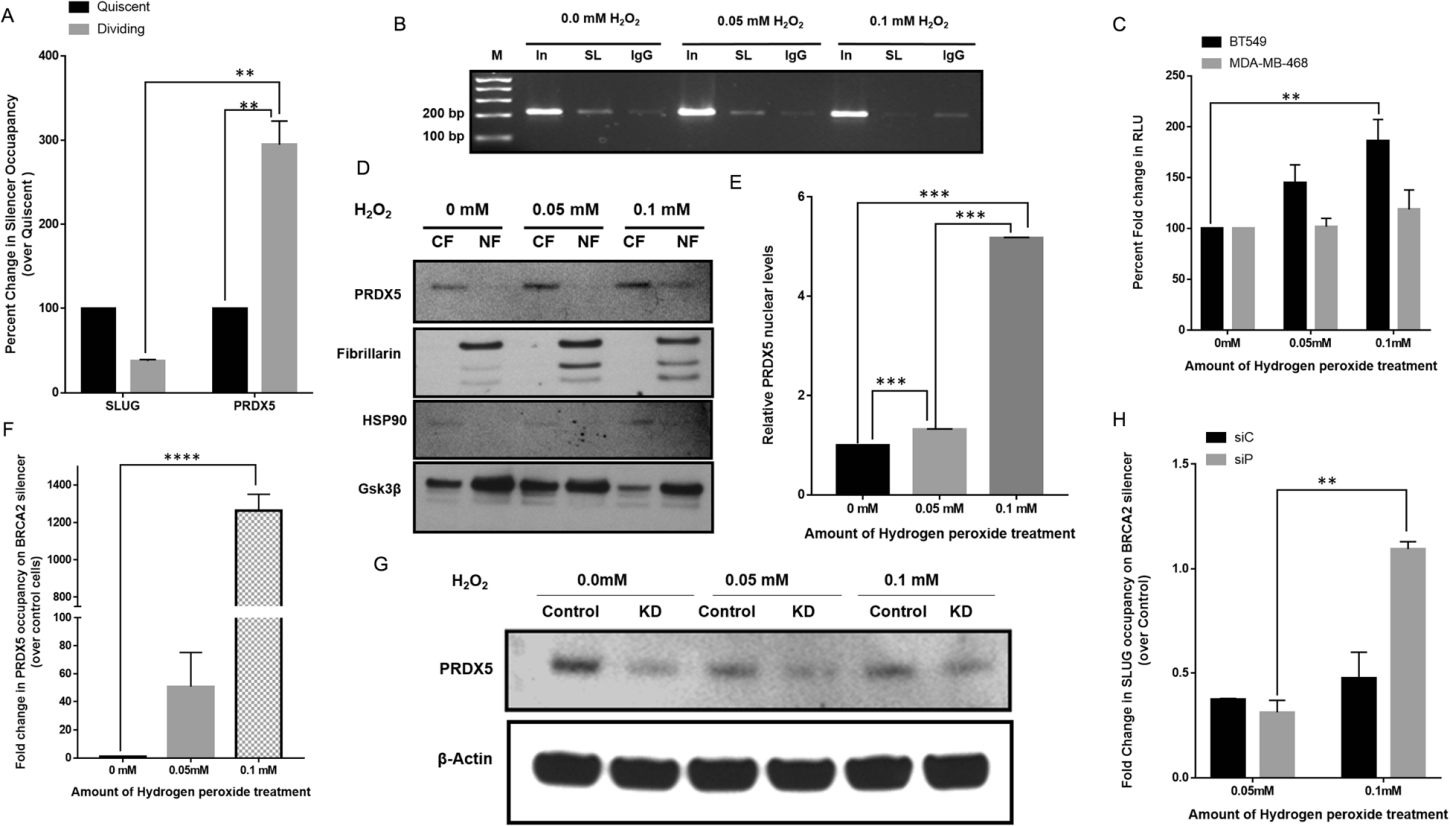
PRDX5 reverses SLUG-mediated *BRCA2* silencing in dividing SLUG-positive BT549 cells. **a** Quantitative ChIP analysis comparing SLUG binding and PRDX5A binding to the *BRCA2* silencer between dividing and quiescent cells. The data were normalized to respective IgG control antibodies and calculated as percent fold change over binding in quiescent cells (designated as 100%). The differences are statistically significant (*p* < 0.001). **b** ChIP assay showing the effect of H_2_O_2_ treatment on SLUG binding to the *BRCA2* silencer. The cells were treated with the respective concentrations of H_2_O_2_ for 24 h prior to chromatin isolation and ChIP assay. M, 1 kb + DNA ladder; SL, pulldown with anti-SLUG antibody; IgG, pulldown with control anti-rabbit IgG; In, Input DNA before pulldown. **c** Dual luciferase reporter assay showing the effect of H_2_O_2_ treatment on the *BRCA2* promoter-silencer activity in SLUG-positive BT549 and SLUG-negative MDA-MB-468 cells. The cells were transfected with the reporter construct and incubated for 16 h. Then, they were exposed to H_2_O_2_ for 24 h, after which the luciferase assay was performed. Untreated cells served as the control (100%) for each cell type. **d** Western blots showing the effect of H_2_O_2_ treatment on nuclear localization of PRDX5. CF, cytosolic fraction; NF, nuclear fraction. Fibrillarin, HSP90, and GSK3β served as a nuclear marker, a cytosolic marker, and the loading control, respectively. **e** Densitometry of western blots in D showing normalized levels of nuclear PRDX5. **f** Quantitative ChIP analysis showing the effect of increasing H_2_O_2_ concentrations on PRDX5 binding to the *BRCA2* silencer. All values were normalized to those from respective IgG-controls and input-DNA. **g** Western blot showing siRNA-mediated knockdown of PRDX5. Control, scrambled_control_siRNA; KD, PRDX5_siRNA. β-actin served as a loading control. **h** Quantitative ChIP analysis of the effect of H_2_O_2_ treatment on SLUG binding to the *BRCA2* silencer with or without PRDX5 knockdown. Results are mean ± SE (*n* = 3). The difference is statistically significant (*p* < 0.0001)

We validated the binding of SLUG and PRDX5A on the *BRCA2* silencer in dividing breast cells through quantitative ChIP analysis. We observed that SLUG binding was reduced by ~ 60% in dividing cells while PRDX5 binding was increased by ~ 50% compared to quiescent cells (Fig. 4a). Since the higher oxidative stress in dividing cells is thought to cause the de-silencing of *BRCA2*-expression in SLUG-positive BC cells, we treated the cells with H_2_O_2_ to mimic the oxidizing environment in dividing cells. We investigated SLUG binding and *BRCA2* promoter activity after H_2_O_2_ treatment (0–0.1 mM). We showed that SLUG binding to the *BRCA2* silencer gradually decreased with increasing H_2_O_2_ concentrations (Fig. 4b). Assessment of *BRCA2* promoter activity in SLUG-positive BT549 and SLUG-negative MDA-MB-468 cells revealed a two-fold increase in promoter activity in BT549 cells treated with 0.1 mM H_2_O_2_ as compared to untreated control (Fig. 4c). However, change in *BRCA2* promoter activity in MDA-MB-468 cells after the same treatment was insignificant. To further validate the oxidative stress induced-derepression of *BRCA2* promoter in SLUG-positive BT549 cells is due to reduced activity of this repressor, we knocked down SLUG using siRNA against SLUG in SLUG-positive BT549 cells (Additional file 1: Figure S1A) and performed dual luciferase assay for BRCA2 promoter-silencer construct in the absence and presence of H_2_O_2_. We observed that in control siRNA treated cells (siControl) there is a gradual increase in the luciferase activity on the treatment of H_2_O_2_ (0–0.1 mM). In the SLUG knocked down cells (siSLUG) those were not treated with H_2_O_2,_ there was a 2-fold increase in luciferase activity compared to siControl cells (Additional file 1: Figure S1C). However, treatment of the SLUG knocked down cells with increasing concentrations of H_2_O_2_ (0–0.1 mM), there was no significant increase in the reporter activity was observed, (Additional file 1: Figure S1C), suggesting that oxidative stress somehow hampered the SLUG repressor acitivity. Interestingly, the two-fold increase in *BRCA2* promoter activity upon increase in oxidative stress mirrored a similar increase in dividing cells shown in Fig. 1d suggesting that increased oxidative stress in replicating cells induces the de-silencing of *BRCA2*-expression by reducing SLUG binding to the silencer.

Next, we investigated if H_2_O_2_ treatment altered the nuclear accumulation of PRDX5A. Immunoblot analysis of the nuclear and cytosolic fractions showed that nuclear accumulation of PRDX5A (~ 18 kDa) increased with increasing H_2_O_2_ concentrations (Fig. 4d, e). Results from our qChIP analysis further showed that PRDX5A binding on the *BRCA2* silencer increased by ~four-fold upon H_2_O_2_ treatment (Fig. 4f) indicating that increased oxidative stress led to nuclear accumulation of PRDX5A, which then competed with SLUG for binding to the *BRCA2* silencer and consequently upregulated *BRCA2*-expression. We knocked down PRDX5A protein levels in BT549 cells using siRNA and performed qChIP analysis for SLUG binding to the *BRCA2* silencer in the presence of H_2_O_2_. PRDX5A proteins levels were significantly reduced in the knockdown cells (Fig. 4g). However, we noticed a slight increase in PRDX5A protein levels in knockdown cells after H_2_O_2_ treatment (0.05–0.1 mM) (Fig. 4g), it has been shown that oxidative stress may stimulate PRDX5A expression [27]. Knockdown cells treated with 0.05 mM of H_2_O_2_ exhibited similar extent of SLUG binding to the *BRCA2* silencer as cells transfected with control siRNA (Fig. 4h). Even though H_2_O_2_ treatment also increased PRDX5A transcription in the siPRDX5 cells, H_2_O_2_ treatment at a higher concentration (0.1 mM) caused an increased in SLUG binding in the siPRDX5 cells compared to control (Fig. 4h), as there is not enough PRDX5 to relive SLUG from silencer region.

### Characterization of the subcellular targeting motifs of PRDX5A

**Fig. 5:**
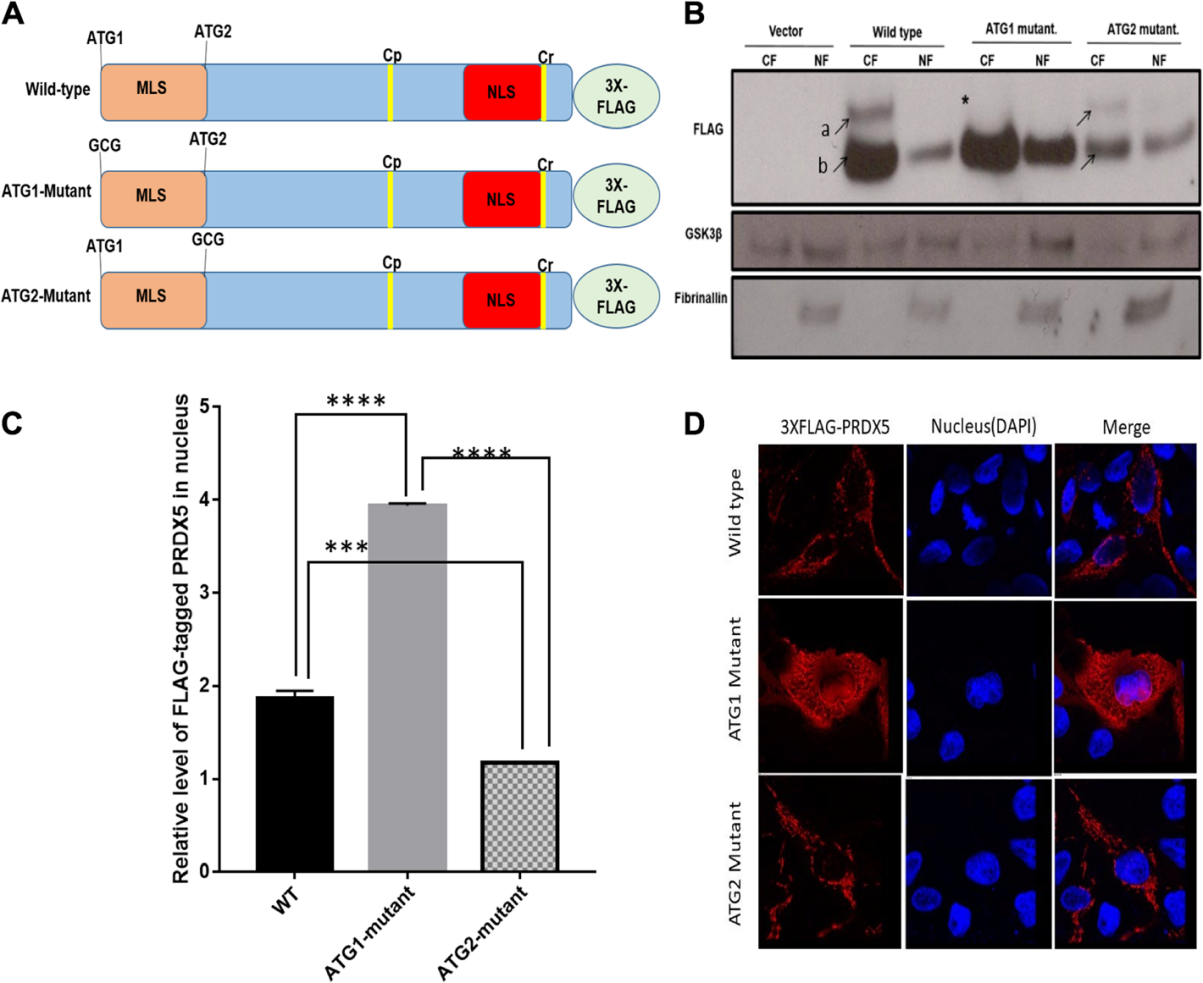
Nuclear PRDX5 originates from the second in-frame ATG codon in the *PDRX5* ORF. **a** Schematics of the PRDX5A constructs in the p3XFLAG-CMV 14 vector. The wildtype ORF with ATG1 and ATG2 was cloned to express a 24-kDa protein FLAG-tagged at the C-terminus. Constructs with mutations at the start codons (ATG1-mutant and ATG2-mutant) were also generated. C_P_ and C_R_ represent codons for active site Cys. MLS, mitochondrial localization signal; NLS, nuclear localization signal; 3X-FLAG, FLAG tag from the vector. **b** Immunoblot showing expression of the recombinant proteins. The wildtype and ATG2-mutant (both ~ 24 kDa, a) existed as precursor proteins. The size of the mature form (~ 18 kDa, b) is the same as that of the protein expressed by the ATG2-mutant construct. Fibrillarin and GSK3β served as a nuclear marker and a loading control, respectively. **c** Western blot quantification for the levels nuclear PRDX5A recombinant proteins. Results are mean ± SE (*n* = 3). The difference is statistically significant (*p* < 0.0001). **d** Immunofluorescence analysis showing nuclear localization of FLAG-tagged PRDX5A (wildtype), ATG1-mutant, and ATG2-mutant in unsynchronized cells. Anti-FLAG M2 antibody was used to detect FLAG-tagged PRDX5A (red) and DAPI was used as a nuclear stain (blue)

To understand what regulates the localization of PRDX5A, we examined its targeting signals. We first verified that the N-terminal MLS (1–50 aa) and the C-terminal NLS (175–203 aa) are functional by attaching these targeting signals to the tomato fluorescent protein. MLS targeted the tomato protein to mitochondria and NLS targeted the tomato lectin to nucleus (Additional file 1: Figure S4 and S5). Therefore, both the MLS and NLS of PRDX5A are functional. Since the nuclear and cytosolic/mitochondrial forms of PRDX5A are of similar size (~ 18 kDa) and the MLS resides between the two in-frame AUGs of the 5′-translated region, we speculated that nuclear PRDX5A is translated from the second start site. To test this, we created two PRDX5A mutants, ATG1-mutant and ATG2-mutant, where either one of the two ATG codons was mutated (Fig. 5a). The wildtype and mutated proteins were FLAG-tagged at the C-terminus. Immunoblot analysis using anti-FLAG antibody detected wildtype PRDX5A-FLAG as two distinct protein bands in the nuclear fraction, marked as ‘a’ (~ 24 kDa) and ‘b’ (~ 18 kDa) in Fig. 5b. The major ~ 18 kDa protein in the post-nuclear fraction (CF) is most likely the matured PRDX5A formed in the mitochondria, where MLS is cleaved off. A minor fraction of the larger protein (~ 24 kDa) could be the precursor form (long, LPRDX5A). Some SPRDX5A was also present in the NF (Fig. 5b). In contrast to wildtype PRDX5A, the ATG1-mutant produced a single protein of ~ 18 kDa in size, indicating that this form of PRDX5A was translated from the second ATG site and lacked the MLS. However, the ATG2 mutant behaved similarly to wildtype PDRX5A and produced both LPRDX5A and SPRDX5A, though at lower levels than the wildtype proteins. Interestingly, we noticed that nuclear accumulation of the ATG1-mutant was 1.5-fold higher than that of the ATG2-mutant and two-fold higher than that of the wildtype protein (Fig. 5b, c). These results suggest that translation from the second ATG site facilitates nuclear accumulation of PRDX5A. Immunofluorescence analysis using confocal microscopy also showed that wildtype PRDX5A and the ATG2-mutant resided primarily in the cytosol, appearing as punctate staining. On the other hand, distribution of the ATG1-mutant is more diffused in the cytosol, and some of the protein was localized at the nucleus (Fig. 5d). Translation of the ATG1-mutant began at the second ATG; hence, the translated product lacked the MLS. As a result, ATG2-mutant appeared more diffuse in the cytosol and was also detected in the nucleus. Therefore, these results indicate that translation initiation from either the first or the second ATG site determines the subcellular localization of PRDX5A.

### Mitronic miR-6855-3p binds to the inter-AUG sequence of the *PRDX5A* transcript and is regulated by oxidative stress

**Fig. 6:**
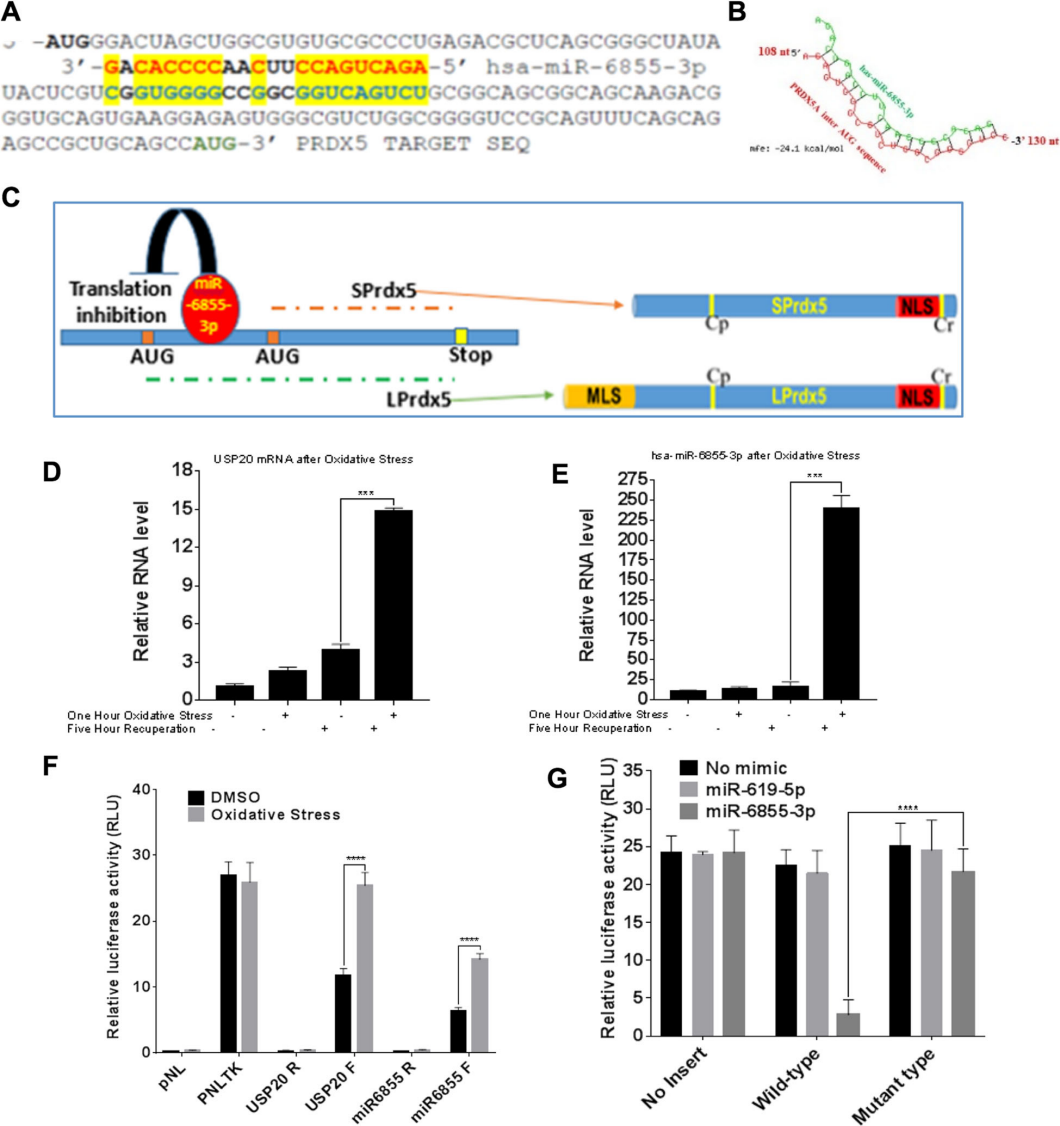
miR-6855 binds to the inter-AUG sequence of the *PRDX5A* transcript and regulates nuclear localization of PRDX5A. **a** Sequence alignment of miR-6855-3p in the 3′-to-5′ orientation with the *PRDX5* mRNA sequence in the 5′-to-3′ orientation to predict miR-6855-3p binding site. **b** The RNA hybrid structure from RNAhybrid analysis between miR-6855-3p and PRDX5-interAUG (54-75 bp) is shown, with minimum free energy; mfe = − 24.1 kcal/mol. **c** Schematic of the binding of miR-6855-3p between the two in-frame start codons of the *PRDX5* mRNA to illustrate the difference between SPRDX5 (lacks MLS) and LPRDX5 (contains MLS). **d** and **e** qPCR to determine relative RNA levels for *USP20* (**d**) and miR-6855-3p (**e**) after 1 h of H_2_O_2_ treatment followed by 5 h of recovery time. **f** Luciferase reporter assay showing the effect of SFP treatment on miR-6855-3p-specific and *USP20*-specific promoters **g** Luciferase reporter assay to determine the effect of miR-6855-3p mimic on the wildtype and mutant inter-AUG sequence of the *PRDX5A* transcript

With the increasing role of redox-regulated miRNA in gene expression regulation and DNA damage/repair pathways we wanted to explore if PRDX5A subcellular localization is driven by a miRNA. To investigate our speculation that choice of ATG is driven by miRNA, we searched for a potential miRNA binding site within the inter-AUG sequence of the *PRDX5A* transcript. Our search in the miRbase database [33] identified hsa-miR6855-3p, which has 80% complementarity to the inter-AUG sequence (54–75 bp) of the *PRDX5A* transcript (Fig. 6a). Therefore, hsa-miR6855-3p could potentially regulate PRDX5A translation. Figure 6b depicts the RNA hybrid secondary structure of mir-6855-3p with PRDX5-inter-AUG. It is a mitronic miRNA that originates from intron 13 of the *USP21 for USP20 and P22* gene on chromosome 9. USP21 for USP20 and P22 is a deubiquitinase that has recently been shown to participate in genome maintenance and repair [34, 35]. We speculated that miR6855-3p binds to the inter-AUG sequence of the *PRDX5A* transcript, thus inhibiting translation of PRDX5A from the first AUG and facilitating translation from the second AUG (Fig. 6c). This translation event yields SPRDX5A that lacks MLS, which localizes to the nucleus. The nuclear SPRDX5A can then regulate *BRCA2* transcription.

To determine the effect of oxidative stress on the expression of miR6855-3p and its host gene *USP21 for USP20 and P22*, we treated BT549 cells with oxidizing agent tBHP. We observed that the levels of *USP21 for USP20 and P22* increased by ~ 15 times in the treated cells compared to the untreated cells (Fig. 6d). tBHP treatment also increased transcription of miR6855-3p by ~ 200 times (Fig. 6e). The discrepancy between the increase in the transcription of miR6855-3p and *USP21 for USP20 and P22* could be due to the presence of an additional promoter that controls the transcription of miR6855-3p, apart from the one shared with *USP21 for USP20 and P22*. To confirm that oxidative stress regulates the activity of the miR6855-3p and *USP21 for USP20 and P22* promoters, we cloned these putative promoter regions into luciferase reporter plasmids. We transfected cells with these plasmids, treated them with oxidizing agent SFP, and measured their luciferase reporter activity. We observed a ~two- to three-time increase in activities for both promoters upon treatment with SFP (Fig. 6f).

To validate miR6855-3p binding to the inter-AUG sequence of the *PRDX5A* transcript, we created reporter constructs using pMIR-REPORT Luciferase by cloning the wildtype and mutant miR6855-3p binding site separately downstream of the reporter gene. We transfected MDA-MB231 BC cells with these plasmids and measured their luciferase activity with and without addition of the miR6855-3p mimic. Non-specific miR-619-5p mimic is used as a negative control. Our data showed that the miR6855-3p mimic inhibited luciferase reporter activity compared to controls (Fig. 6g). Moreover, the miR6855-3p mimic only inhibited the reporter activity when the wildtype *PRDX5* inter-AUG sequence was attached downstream of the reporter (Fig. 6g). The mutated inter-AUG sequence did not cause a significant change in reporter activity with either miRNA mimics. These results suggest that miR6855-3p plays a role in regulating alternative translation of the *PRDX5* transcript.

### Presence of miR-6855-3p increases nuclear accumulation of SPRDX5A

**Fig. 7:**
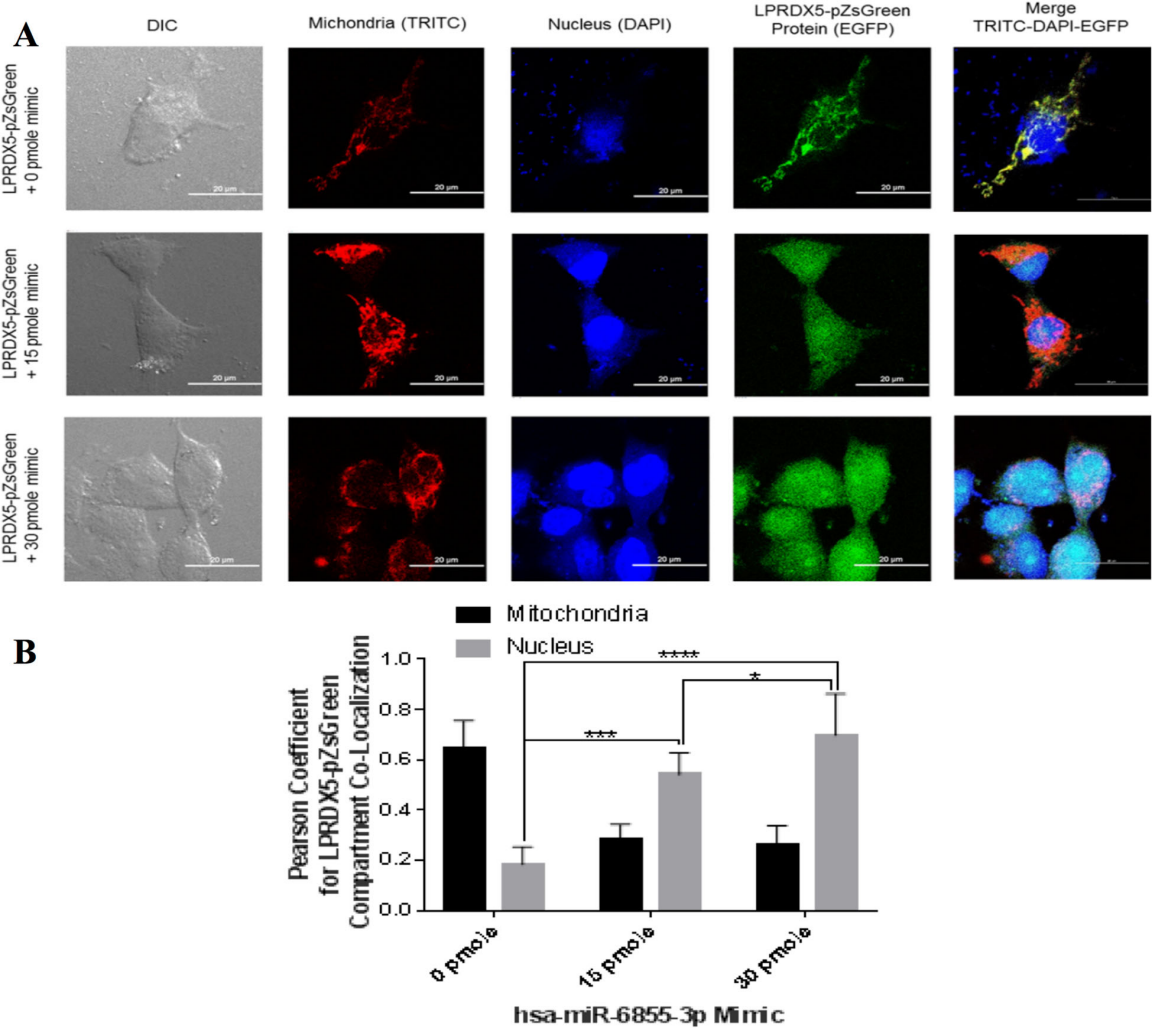
miR-6855-3p increases the nuclear accumulation of SPRDX5A. Full-length LPRDX5 cDNA was cloned into the pZsGreen vector to generate a C-terminal GFP-tagged LPRDX5 protein (LPRDX5-pZsGreen). **a** Confocal microscopy analysis of LPRDX5-pZsGreen in the absence or presence of miR6855-mimic. **b** Images such as those in **a** were used to obtain the Pearson coefficients for subcellular colocalization to determine colocalization of LPRDX5-pZsGreen with either the mitochondria or the nucleus

Next, we investigated if addition of the miR-6855-3p mimic could alter the subcellular location of PRDX5A in SLUG-positive BC cells. To do this, we cotransfected LPRDX5A-pZsGreen plasmid and treated them with the miR6855-3p mimic at different concentrations (0–30 pmole/ml). Using confocal imaging, we showed that without the miR6855-3p mimic, PRDX5A-pZsGFP localized primarily to the mitochondria (Fig. 7a, b). In the same experimental condition, some PRDX5A-pZsGFP also localized to the nucleus (Fig. 7a, b). However, when the cells were treated with 15 pmole miR6855-3p mimic, nuclear localization of PRDX5A-pZsGFP increased significantly (Fig. 7a, b). This increase coincided with a significant decrease in mitochondrial localization of PRDX5A-pZsGFP. At a higher concentration of miR6855-3p mimics (30 pmole), almost 80% of PRDX5A-pZsGFP localized at the nucleus. These results show that the miR6855-3p mimic increased nuclear localization of PRDX5A.

## Discussion

Here, we demonstrate a novel and unique mechanism for oxidative stress-induced de-silencing of *BRCA2*-expression by PRDX5A. Our results demonstrate that higher oxidative stress in replicating SLUG-positive BC cells upregulates the expression of a unique mitronic miRNA miR6855-3p. miR6855-3p binds to the inter-AUG sequence of the *PRDX5A* transcript and facilitates translation initiation from the second-AUG codon to produce SPRDX5A that lacks the MLS. Consequently, SPRDX5A accumulates in the nucleus and de-silences *BRCA2*-expression by displacing the SLUG from the *BRCA2* silencer.

Multiple studies have indicated that BRCA2 dysfunction causes various cancers. Dividing cells express BRCA2 to protect the genome from accumulating errors caused by double-stranded break (DSB) repairs through non-homologous end joining to prevent oncogenesis [36]. Therefore, understanding the cell cycle-mediated regulation of *BRCA2*-expression is critical for evaluating the etiology of human BC.

We previously reported that cell cycle-dependent regulation of *BRCA2-*expression in SLUG-positive BC cells occurs via chromatin remodeling mediated by SLUG through the E2-box and Alu repeats containing silencer region [13, 15]. Through this study, we uncover a novel and unique mechanism that reverses the SLUG-induced silencing of *BRCA2*-expression in SLUG-positive BC cells, and provide evidence that the B-box binding protein PRDX5A mediates this reversal.

First, we observed that nuclear localization and recruitment of PRDX5A on the *BRCA2* silencer were increased in dividing cells. Next, we determined that increased nuclear PRDX5A binding to the silencer caused the de-silencing of *BRCA2*-expression in SLUG-positive BC cells. Finally, we demonstrated that the *PRDX5A* mRNA possesses two in-frame AUG codons, and that translation of nuclear PRDX5A (SPRDX5A) begins at the second AUG site. This translation event is mediated by redox-responsive hsa-miR-6855-3p that binds to the inter-AUG sequence.

Since dividing cells experience higher oxidative stress than non-dividing cells, we also examined how oxidative stress affects the expression and nuclear localization of PRDX5A as well as the de-silencing of *BRCA2*-expression. PRDX5 is a cytoprotective antioxidant enzyme that counteracts endogenous or exogenous peroxide attacks rather than acting as a redox sensor [24, 26]. PRDX5A localizes to the mitochondria, cytoplasm, peroxisomes and nucleus [24, 25, 37, 38]. Higher PRDX5A levels are detected in aggressive Hodgkin-lymphomas, malignant-mesothelioma, breast-carcinoma, ovarian-carcinoma, and thyroid cancer [24]. It has been shown that in lung cancer cells, PRDX5A plays a role in DSB repair caused by etoposide treatment and that PRDX5A-mediated DSB repair does not depend on the enzymatic activity of PRDX5A [38]. PRDX5A has also been shown to localize to the Cajal bodies in the nucleus, which are sites of active transcription [38–40]. Immunofluorescence analysis has shown that PRDX5A colocalizes with p80-coilin, the main component of Cajal bodies [38].

While studying the involvement of PRDX5A in regulating *BRCA2*-expression, we analyzed the status of PRDX5A in BC cells. We observed that the expression of BRCA2 and PRDX5A protein increased in dividing cells as well as in cells treated with H_2_O_2_. We also observed an increase in levels of nuclear PRDX5A in dividing cells compared to non-dividing cells, and in response to increasing H_2_O_2_ concentrations. We also observed that the de-silencing of *BRCA2*-expression occurred due to the loss of SLUG binding at the silencer. Using qChIP analysis, we verified that the loss of SLUG binding at the *BRCA2* silencer correlated with increased levels of nuclear PRDX5A and its binding to the *BRCA2* silencer. We speculate that the physical proximity of the E2-box and B-box brings SLUG and PRDX5A close together, allowing PDRX5A to perhaps oxidize the zinc fingers in SLUG. This oxidation displaces SLUG from the silencer.

As there are multiple PRDX5 isoforms, we wanted to determine which isoform is present in the nucleus and responsible for de-silencing *BRCA2*-expression. We observed that PRDX5A isoform is the stable isoform and results from our mutational analysis of the two in-frame AUG codons at the 5′-translated region of the *PRDX5A* transcript indicate that alternative translation initiation results in two different forms of PRDX5A which localize to different subcellular compartments.

Recent studies have shed light on the role of miRNAs as trans-acting factors that post-transcriptionally regulate protein expression [41–43]. It has been shown that in addition to the 3′UTR, the coding region of mRNAs can also serve as miRNA binding sites [44–46]. However, since all conventional miRNA binding site prediction algorithms focus on the 3′UTR, we conducted a manual search for miRNA candidates that can bind to the inter-AUG sequence of PRDX5A. Our search in the miRbase database [33] revealed hsa-miR-6855-3p as a promising candidate for binding the inter-AUG sequence, with minimum free energy of hybridization (mfe) of − 24.1 kcal/mol as calculated by the RNAhybrid program [47]. The 3′UTR of the *PRDX5A* mRNA does not contain any binding site for miR-6855-3p. Interestingly, miR-6855-3p is a mitronic miRNA that is nested in intron 13 of *USP21 for USP20 and P22* on chromosome 9. USP21 for USP20 and P22 is responsible for the expression of hypoxia-inducible factor (HIF)-1alpha -controlled genes, many of which play a role in oncogenesis. Therefore, concomitant increase in its levels promotes cancer progression [48]. We observed that the promoter activity and transcript levels of USP21 for USP20 and P22 and miR6855 increased at higher oxidative state. Using mutational analysis we validate that miRNA-6855-3p can bind to the inter-AUG sequence of the *PRDX5A* mRNA. Increases in nuclear-PRDX5A and decrease in the mitochondrial-PRDX5A was observed through confocal microscopy, in response to increasing amounts of miR-6855-3p mimic. Taken together, our findings show that higher oxidative stress results in higher miR-6855-3p levels. miR-6855-3p binds to the inter-AUG sequence in the *PRDX5A* mRNA and renders translation from the second AUG codon preferable. This results in higher levels of SPRDX5A, which localizes to the nucleus. Increased levels of SPRDX5A in the nucleus reverse SLUG-mediated silencing of *BRCA2*-expression.

Further investigation is warranted to determine how miR-6855-3p influences the translation machinery to skip the first AUG and instead begin translation from the second AUG of the *PRDX5A* transcript. Nonetheless, the miRNA-mediated, alternative translation of the *PRDX5A* transcript represents a novel form post-transcriptional regulation of gene expression by miRNAs. It indicates that miRNAs not only regulate total protein turnover, but also promote the synthesis of different protein from the same mRNA which, can have different effects on various cellular processes.

Cancer cells continuously experience higher ROS-production compared to normal cells; yet, they can protect themselves from ROS-mediated apoptosis and damage by upregulating free radical scavenging enzymes. This phenomenon may underlie chemo- and radio-resistance as these treatments rely on ROS-production. Higher levels of PRDX5 in hormone-receptor-negative tumors have been associated with tumor-node metastasis, higher tumor volumes, and shorter survival [49]. Higher levels of BRCA2 in this type of tumors also correlate with poor survival outcomes of BC patients [50]. Our current study uncovers a link between increased PRDX5A levels and de-silencing of *BRCA2*-expression under oxidative stress.

To the best of our knowledge, we are the first to report a mechanistic relationship between increased oxidation stress and increased *BRCA2*-expression in BC cells mediated by PRDX5A. This de-silencing mechanism is also applicable to other redox-responsive genes that possess PRDX5A binding site.

## Conclusion

Here, we report a novel and unique mechanism for oxidative stress-induced de-silencing of *BRCA2*-expression by PRDX5A. Our results demonstrate that increased oxidative stress in replicating SLUG-positive BC cells upregulates the expression of miR-6855-3p, which binds the inter-AUG region of the *PRDX5A* transcript and promotes translation from the second-AUG codon. This translation yields SPRDX5A, without MLS and it accumulates in nucleus and de-silences *BRCA2*-expression by displacing the SLUG from the *BRCA2*-silencer.

## Supplementary information

**Additional file 1: Table S1.** Sequences of the oligonucleotides used in this study. **Figure S1.** SLUG is needed for the silencer mediated repression of BRCA2 gene promoter in SLUG-positive BT549 cells. **Figure S2.** Dividing cells have higher oxidation state compared to no-dividing (quiescent). **Figure S3.** Protein translated from the splice variant PRDX5A is the only stable form. **Figure S4.** Mitochondrial localization signal is enough to take the protein to mitochondria. **Figure S5.** Nuclear localization signal is enough to take the protein to the nucleus.

## Data Availability

All data generated or analyzed during this study are included either in this article or in the supplementary Materials and Methods, Tables, Figures and Figure Legends files.

## Abbreviations

BC: breast cancer
ChIP: Chromatin immune pulldown
GAPDH: glyceraldehyde-3-phosphate dehydrogenase
GSK3β: glycogen synthase kinase 3 beta
H_2_O_2_: Hydrogen peroxide
HSP90: Heat shock protein 90
MLS: Mitochondrial localization signal
NLS: Nuclear localization signal
ORF: open reading frame
qPCR: Real time quantitative PCR
RT-PCR: Reverse transcriptase PCR
SFP: sulforaphane
tBHP: ter-butyl hydrogen peroxide
VDAC1: Voltage dependent anion channel 1
