# Supplementary material for: The role of the redox/miR-6855-3p/PRDX5A axis in reversing SLUG-mediated BRCA2 silencing in breast cancer cells
Source: Cell Commun Signal. 2020 Jan 27;18:15. doi: 10.1186/s12964-019-0493-5 (PMC6986021; doi:10.1186/s12964-019-0493-5)
Supplement: Supplementary file 1 — Additional file 1: Table S1. Sequences of the oligonucleotides used in this study. Figure S1. SLUG is needed for the silencer mediated repression of BRCA2 gene promoter in SLUG-positive BT549 cells. Figure S2. Dividing cells have higher oxidation state compared to no-dividing (quiescent). Figure S3. Protein translated from the splice variant PRDX5A is the only stable form. Figure S4. Mitochondrial localization signal is enough to take the protein to mitochondria. Figure S5. Nuclear localization signal is enough to take the protein to the nucleus. [file 12964_2019_493_MOESM1_ESM.docx]

**Additional file 1: Supplementary information**

**Table S1.** Sequences of the oligonucleotides used in this study.

| **Primer Name** | **Sequence (5’ to 3’)** | **Purpose** |
| --- | --- | --- |
| P1 | GCGAGAAGAGAACACACA | Forward primer from BRCA2 Promoter |
| P2 | GCAAGATGGGCCGGGTGT | Forward primer for BRCA2 Silencer |
| P3 | CCA GGGTGTGGTTCTC | Reverse primer for BRCA2 Silencer |
| P4 | ATCGATATGGGACTAGCTGGCGTGTGCG | PRDX5 ORF forward primer with Cla1 site |
| P5 | GGATCCGAGCTGTGAGATGATATTGGG | PRDX5 ORF reverse primer with BamHI site |
| P6 | GGATCCATGGGACTAGCTGGCGTGTGCG | PRDX5 ORF forward primer with BamHI site |
| P7 | AAGCTTGAGCTGTGAGATGATATTGGG | PRDX5 ORF reverse primer with HindIII site |
| P8 | CGGCCGCGAATTCATCGATATCGGACTAGCTGG | Site directed mutation of ATG1 |
| P9 | CCAGCTAGTCCGATATCGATGAATTCGCGGCCG | Site directed mutation of ATG1 |
| P10 | GAGCCGCTGCAGCCGCGGCCCCAATCAAGG | Site directed mutation of ATG2 |
| P11 | CCTTGATTGGGGCCGCGGCTGCAGCGGCTC | Site directed mutation of ATG2 |
| P12 | CGAACTAGTATGGGACTAGCTGGCGTGTGC | Forward primer for PRDX5A inter AUG sequence with Spe1 site |
| P13 | GGAAAGCTTGGCTGCAGCGGCTCTGC | Reverse primer for PRDX5A inter AUG sequence with HindIII site |
| P14 | GTGGCACCCCCCCGCCCAGTCAGAGCGGCAGCGGCAGCAAGACGG | SOE primer for PRDX5A interAUG miR6855 mutation |
| P15 | TCTGACTGGGCGGGGGGGTGCCACGAGTATATAGCCCGCTGAG | SOE primer for PRDX5A interAUG miR6855 mutation |
| P16 | CTCAAGCTTACCATGGGACTAGCTGGCGTGTGC | MLS-forward primer with HindIII site |
| P17 | CGGTGGATCCCGGGCGGCTGCAGCGGCTCTGC | MLS-reverse primer with BamHI site |
| P18 | CAAGCTTCGCGACGTCTCAAGAGGTTCTC | NLS-forward primer with HindIII site |
| P19 | GGTGGATCCGGTCATGAGGCCTGTGCCATCTGG | NLS-reverse primer with BamHI site |
| P20 | AGACTCGAGGGTTAAACATTTGCAAAGAACG | Forward primer with XhoI site for USP20 promoter amplification |
| P21 | AGGAAGCTTAACTCACCTGCACTCGCAACTGC | Reverse primer with HindIII site for USP20 promoter amplification |
| P22 | CACAAGCTTACTAGACCCGAATGACAGT | Forward primer with HindIII site for miR6855 promoter amplification |
| P23 | CACAAGCTTAGGTGAGGGGCAGAGGGACTAC | Reverse primer with HindIII site for miR6855 promoter amplification |
| P24 | AGACTGACCTTCAACCCCACAG | miR6855-qRTPCR |
| P25 | GCTCAGCGGGCTATATACT | PRDX5 RTPCR Forward |
| P26 | ACTATGCCATCCTGTACCAC | PRDX5 RTPCR Reverse |
| P27 | CCTCACCTTGACTCCATAGGA | USP20 qRTPCR Forward |
| P28 | CCCATAGGTTTGGTCCGG | USP20 qRTPCR Reverse |
| P29 | CATGTTCGTCATGGGTGTGAACCA | GAPDH qRTPCR Forward |
| P30 | AGTGATGGCATGGACTGTGGTCAT | GAPDH qRTPCR Reverse |
| P31 | GCTCGTCGTCGACAACGGCTC | βActin qRTPCR Forward |
| P32 | GCTCGTCGTCGACAACGGCTC | βActin qRTPCR Reverse |

**
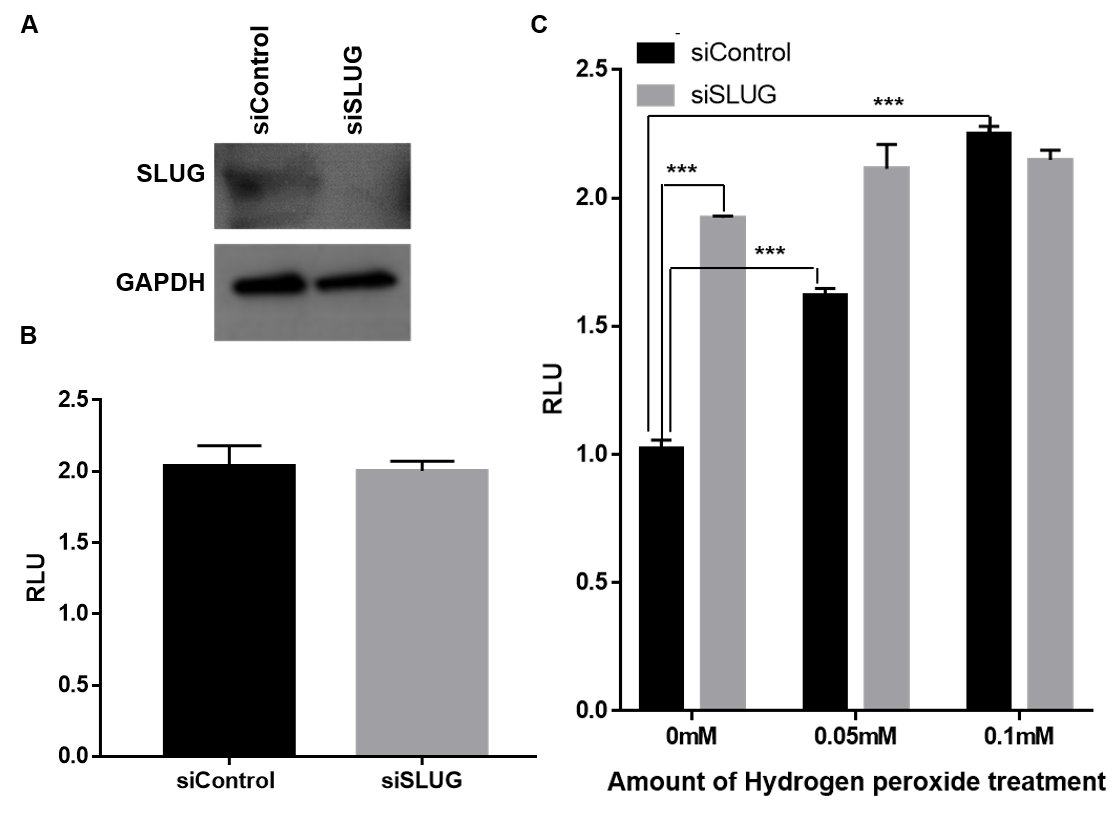
**

**Figure S1: SLUG is needed for the silencer mediated repression of BRCA2 gene promoter in SLUG-positive BT549 cells**. **A.** Western blot showing a decrease in the SLUG protein levels in BT549 cells treated with SLUG siRNA compared to scrambled control. BT549 cells were treated with 100 ηM of either SLUG siRNA (siSLUG) or scrambled control siRNA (siControl) for 24 hrs. SLUG siRNA was procured from Ambion, lnc, Huston, TX (catalog# 4390824) [13]. **B**. Dual Luciferase reporter assay showing the effect of SLUG knock down on the activity human BRCA2 gene promoter (-187 to +310) construct lacking the silencer region in pRL-null vector [14]. The siRNA treated BT549 cells were transfected with the reporter constructs and dual luciferase assay was done after 24 h, of treatment. **C**. Dual luciferase reporter assay showing the effect of H_2_O_2_ treatment on the *BRCA2* promoter-silencer (pRL-PS) activity in BT549 after siRNA mediated SLUG knockdown. The siRNA treated BT549 cells were transfected with the reporter constructs and incubated for 16 h. Then, the cells were exposed to different concentration of H_2_O_2_ for 24 h, after which the luciferase assay was performed. Empty vector (pRL-null) transfected cells served as the control for each treatment.


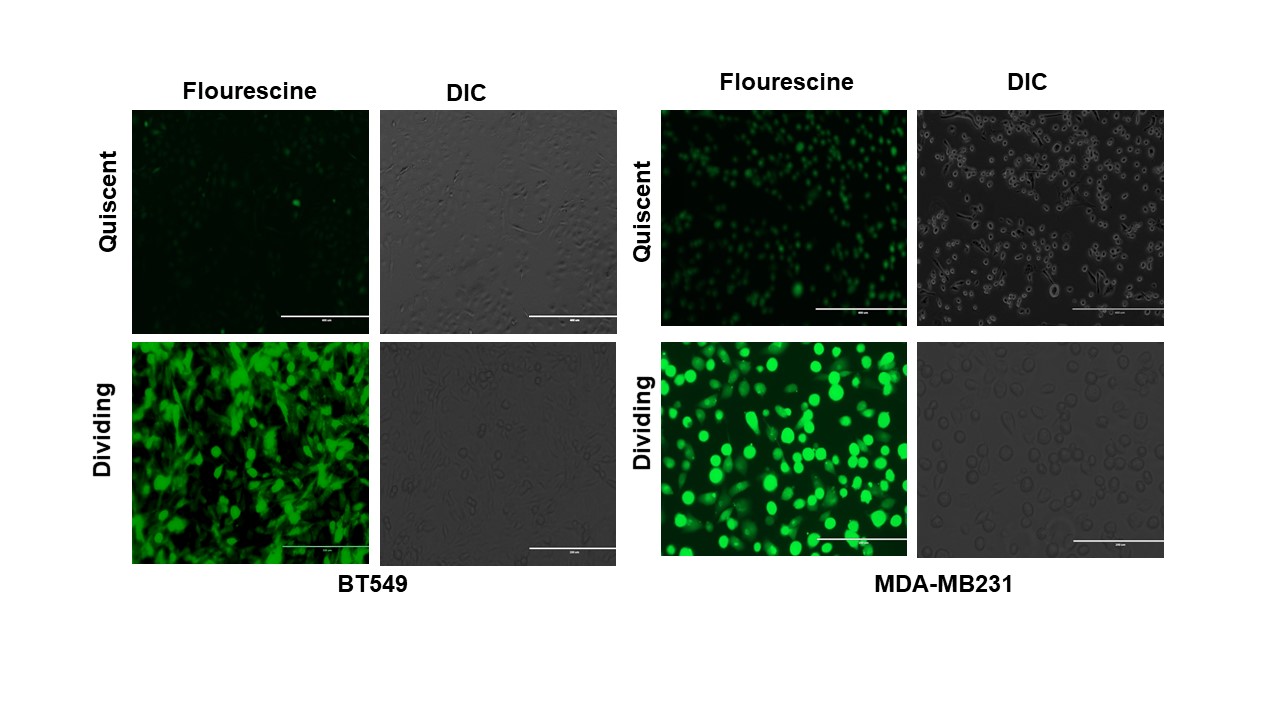


**Figure S2: Dividing cells have higher oxidation state compared to no-dividing (quiescent).** BT549 and MDA-MB231 cells were treated with the DCFDA (2’,7’ –dichlorofluorescein diacetate) for 30 min in growth medium post synchronization and captured immediately after washing the cells through fluorescence microscope. Flourescine: DCFDA converted to DCF and DIC: phase contrast. The brighter the green higher is the oxidation state.
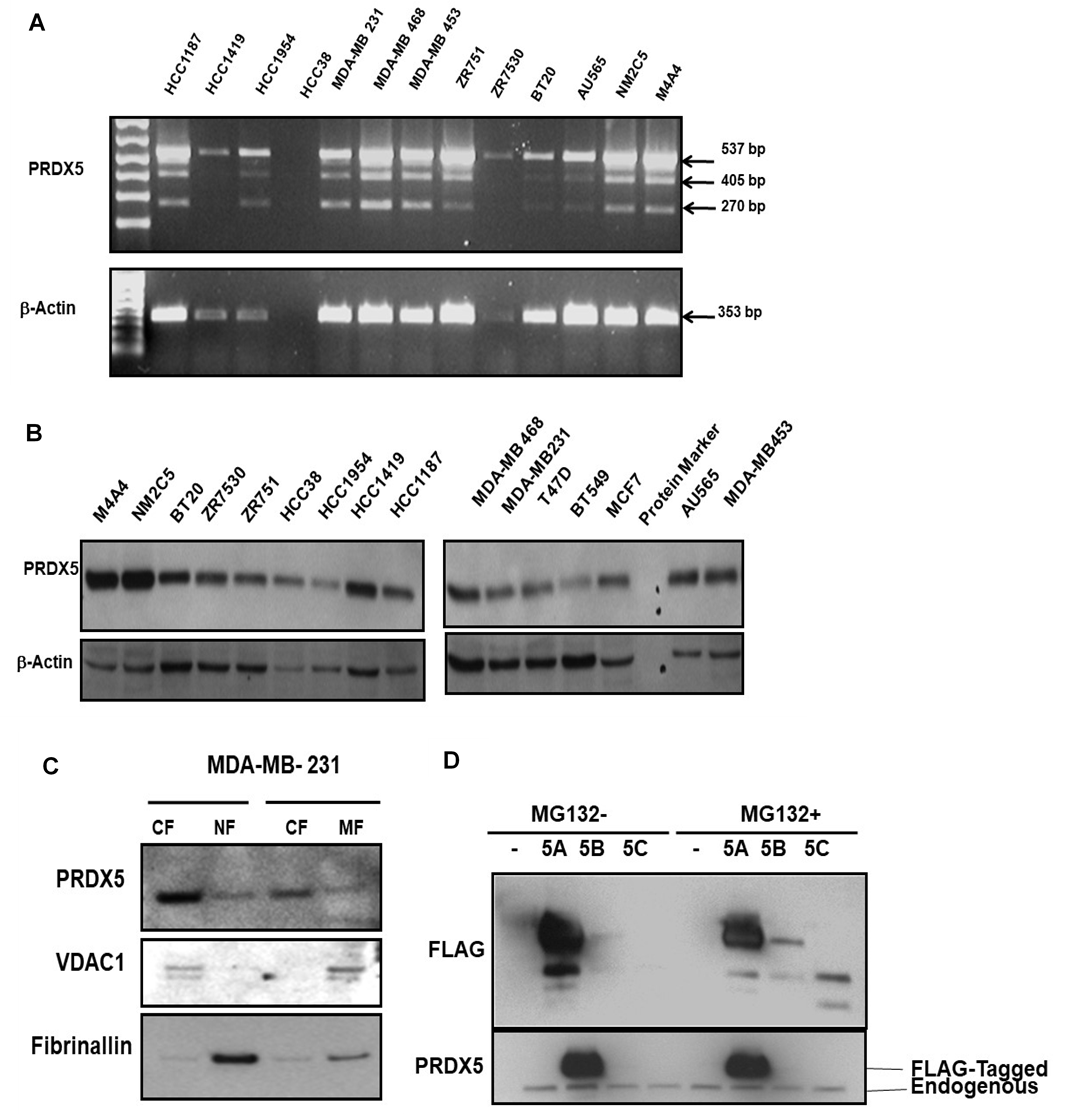


**Figure S3: Protein translated from the splice variant PRDX5A is the only stable form**. **A.** End point RT-PCR showing relative levels of PRDX5A, PRDX5B and PRDX5C mRNA in different breast cancer cell lines*. {*Amplified with the forward primer from Exon 1 (151-169 bp) and reverse primer from exon 4 (669-688 bp PRDX5A); 537-556 bp (PRDX5B) and 402-421 bp(PRDX5C)]}. Expected amplicon size PRDX5A=538 bp, PRDX5C=405 bp and PRDX5C=270 bp. **B**. Western blot analysis showing the PRDX5 protein, ~18KDa in different breast cancer cell lines using the monoclonal antiPRDX5 antibody raised against the C-terminal of protein from BD Biosineces. **C**. Western blot showing the subcellular localization of the PRDX 5 protein in MDA-MB 231 human breast cancer cell by immunoblot analysis. CF: cytosolic fraction, NF: nuclear fraction and MF: mitochondrial fraction. **D**. immunoblot analysis showing the effect of the proteasomal inhibitor MG132 on the stability of the ectopically expressed FLAG-tagged three different splice variants of PRDX5.

**
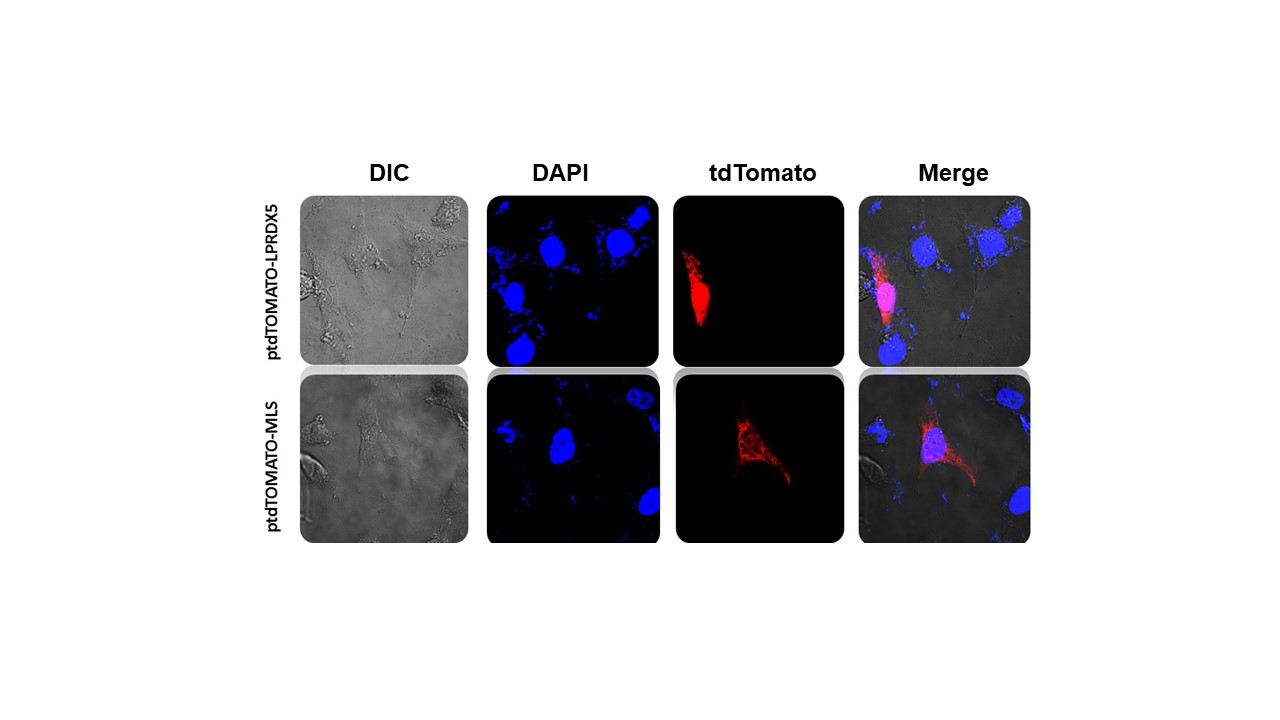
**

**Figure S4: Mitochondrial localization signal is enough to take the protein to mitochondria**. Data showing immunofluorescence confocal microscopy for the localization of tdTomato to mitochondria as seen by reticulate tdTomato staining in MLS-tdTomato construct in transiently transfected BT549 BC cells. The corresponding nucleotide sequence for N-terminal 50 amino acids (MGLAGVCALRRSAGYILVGGAGGQSAAAAARRCSEGEWASGGVRSFSRAAAA) were tagged to the N-terminal of tdTomato and used as MLS.


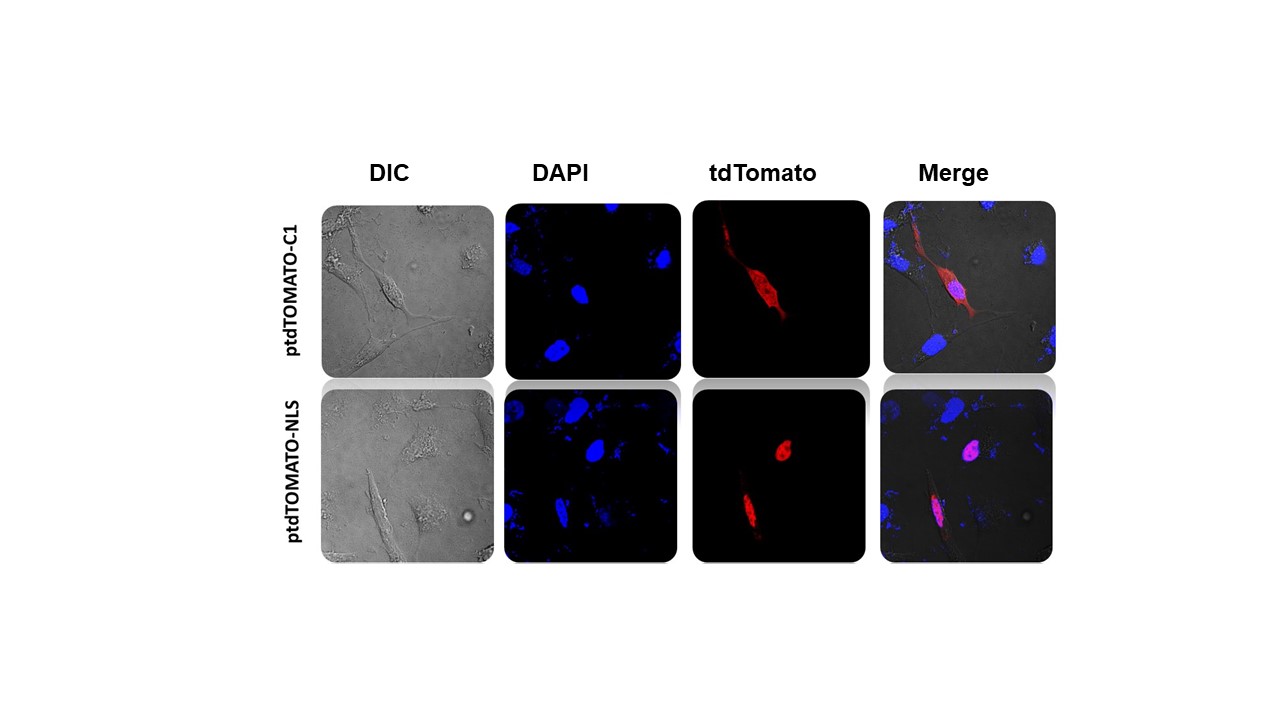


**Figure S5: Nuclear localization signal is enough to take the protein to the nucleus**. Data showing immunofluorescence confocal microscopy for the localization of tdTomato to nucleus as seen in merge with DAPI and tdTomato colocalized. The corresponding nucleotide sequence for amino acids (RRLKRFSMVVQDGIVKALNVEPDGTGLT) from 175 to 203 amino acids of PRDX5A were cloned in ptdTomato-C1, to produce on the N-terminal-tagged tdTomato.
